# The role of childrens’ vaccination for COVID-19—Pareto-optimal allocations of vaccines

**DOI:** 10.1371/journal.pcbi.1009872

**Published:** 2022-02-25

**Authors:** Nir Gavish, Guy Katriel

**Affiliations:** 1 Faculty of Mathematics, Technion - IIT, Haifa, Israel; 2 Department of Applied Mathematics, ORT Braude College of Engineering, Karmiel, Israel; Johns Hopkins University, UNITED STATES

## Abstract

COVID-19 vaccines have been approved for children of age five and older in many countries. However, there is an ongoing debate as to whether children should be vaccinated and at what priority. In this work, we use mathematical modeling and optimization to study how vaccine allocations to different age groups effect epidemic outcomes. In particular, we consider the effect of extending vaccination campaigns to include the vaccination of children. When vaccine availability is limited, we consider Pareto-optimal allocations with respect to competing measures of the number of infections and mortality and systematically study the trade-offs among them. In the scenarios considered, when some weight is given to the number of infections, we find that it is optimal to allocate vaccines to adolescents in the age group 10-19, even when they are assumed to be less susceptible than adults. We further find that age group 0-9 is included in the optimal allocation for sufficiently high values of the basic reproduction number.

## Author summary

The management of a vaccination campaign requires constant evaluation, and, as conditions change, redesign of the campaign for the future. One example of a change that leads to a decision point is the extension of vaccine eligibility to broader segments of the population. Indeed, one of the questions public health experts and policymakers confront is whether children of age 5 and older should be vaccinated against COVID-19 and at what priority. In this work, we use mathematical and computational methods to study this question in a systematic, quantitative way, by considering optimal outcomes, under different criteria for optimality, that can be achieved with and without vaccination of children, under conditions of limited vaccine availability. To explore tradeoffs among different goals, such as reducing the number of infections and reducing mortality, we use the the idea of Pareto optimization. Our results show that, under a broad range of conditions, optimal vaccine allocations include vaccination of age group 10–19, while the population-level benefits of vaccination of age group 0–9 depend on the reproductive number and vaccine availability.

## 1 Introduction

Vaccination campaigns have been rolling out in many countries since the approval of the first vaccine for the prevention of coronavirus disease 2019 (COVID-19) in December 2020. These ongoing vaccination campaigns are subject to changing conditions, e.g., the emergence of new variants, the gradual extension of age eligibility, varying levels of hesitancy, supply issues, waning of vaccine induced immunity and accumulation of convalescent immunity. Furthermore, improved pharmaceutical treatment for those infected [1, 2] and accumulation of evidence of post-COVID conditions [3] may gradually shift the goals of vaccination campaigns from the direct and indirect protection of those at risk of developing severe outcomes (mortality minimizing strategies) to the reduction of overall infections. Accordingly, the management of a vaccination campaign requires constant evaluation and, as conditions change, redesign, while considering up-to-date goals and the optimal ways to achieve them.

One example of a change that leads to a decision point regarding the management of a vaccination campaign is the extension of vaccine eligibility to broader segments of the population. Recently vaccines have been approved for children of age five and older following evidence that the known and potential benefits of the vaccine in individuals down to 5 years of age outweigh the known and potential risks [4]. Nevertheless, many countries debate whether they should extend their vaccination campaigns to include the vaccination of children [5] given that vaccination efforts around the world are being restrained by a shortage of doses [6, 7]. Under limitations on vaccine availability, the vaccination of one age group involves an opportunity cost in vaccinating fewer individuals of another. In other cases, mass vaccination of children may involve opportunity costs related to logistic efforts or public awareness campaigns that could have been focused on different age groups. At the time of submission, the US has decided to vaccinate all eligible children, and some European countries recently decided to administer coronavirus vaccinations to children of age 5–11 [8, 9]. This debate is likely to reemerge in countries that have already decided to vaccinate children when considering booster or lineage-adapted vaccines.

A key question under consideration in this debate is whether and to what extent vaccination of children will enhance the effectiveness of a vaccination campaign at the population level [5, 10–12]. When attempting to assess the relative merits of allocating vaccines to the younger age groups, one must take into account the epidemiological characteristics of COVID-19 concerning these groups, and these seem to point in opposing directions. On the one hand, children infected with COVID-19 rarely develop severe disease [13, 14]. In addition, it has been estimated that children’s susceptibility to infection by SARS-CoV-19 is lower than that of adults [15–17]. On the other hand, children are a relatively large age group that tends to interact more intensively than other age groups. A large epidemic outbreak among children is likely to spread to older age groups, and risk vaccinated and non-vaccinated adults, so that vaccination of children indirectly protects individuals of other age groups [18], who are at greater risk of severe outcomes. These considerations suggest that, in the case of COVID-19, quantifying the *indirect* effect of vaccinating the younger age groups in protecting the older, more vulnerable age groups is essential for evaluating the benefits of allocating vaccines to children and adolescents. Ethicists have been debating whether vaccinating children is justified, under the appropriate circumstances, even if the primary aim of doing so is to protect the older age groups [19, 20]. However, under conditions of limited availibility, this debate is only relevant if indeed allocating vaccines to children enables achieving better outcomes than allocating them to older age groups.

The goal of this work is to contribute to the discussion on childrens’ vaccination by systematically exploring age-dependent allocation of vaccines with the aim maximizing the population-level impact of a vaccination campaign. We present a mathematical model to study the effect of demography, age-based social interaction structure, and vaccine efficacy on the optimal post-vaccination outcomes that can be achieved by suitable allocation of vaccines in the context of the COVID-19 pandemic. The model can be applied at any stage of the vaccination campaign, taking into account existing convalescent and vaccine-induced immunity. Our study is motivated by two questions:

1. How essential is the vaccination of children and youths to achieving herd immunity? Specifically, what are the prospects for achieving herd immunity with the aid of vaccination, assuming vaccine allocation is performed optimally, and what are the age-dependent vaccine allocations which will achieve herd immunity with minimal vaccination coverage?
2. What is the population-level impact of vaccination of children in case herd immunity by vaccination cannot be attained? What are the optimal outcomes—according to different possible measures—that can be achieved, and what are the age-dependent vaccine allocations that will achieve them?

To address these questions, we compare scenarios in which all age groups are eligible, by policy, for vaccination, to scenarios in which vaccination is restricted only to those over 10, or only to those over 20. When children *can* be vaccinated, we wish to determine whether it is *optimal* to do so, under conditions of limited vaccine availability. When children *cannot* be vaccinated, we seek to evaluate whether and to what extent the optimal achievable outcomes are degraded relative to the case in which children can be vaccinated. Several model-based studies address the question of optimal vaccine allocations for SARS-Cov-19. Moore et al. [21] studied vaccination strategies for COVID to minimize future deaths or quality-adjusted life year losses. This study was conducted at an early stage of the pandemic when uncertainty regarding the vaccines was high. Bubar et al. [22] and Isalem et al. [23] focus on the design of a vaccination campaign as it competes with the spread of infection. Matrajt et al. [24] as well as Meehan et al. [25], used an age-stratified model to study the consequences of vaccine effectiveness and population coverage on the optimal vaccine allocation. These works show that when available vaccine coverage is relatively low, mortality-minimizing vaccine allocations prioritize the elderly, while for sufficiently high coverage, the mortality-minimizing vaccine allocations are those that prioritize younger populations who are the drivers of the epidemic. Vaccine allocations that are optimized according to other criteria which are correlated with mortality, e.g., ICU peak, give rise to qualitatively similar patterns, but the point of transition between vaccination of the young and vaccination of the elderly varies.

In this work, we investigate outcomes of a vaccination campaign in the medium-term range after the vaccination effort has ended. Our work is complementary to the above-mentioned studies [21, 22, 24, 25] in both its focus and methods used. We specifically address the issue of restrictions on the eligibility of children and adolescents and quantify the consequences of choosing not to vaccinate them.

An additional feature of this work is the simultaneous consideration of several objectives of a vaccination campaign by employing the concept of Pareto-optimality. Vaccination policies are commonly optimized for a single measure such as mortality, the number of infections, quality-adjusted life year losses, or hospitalizations [21, 22, 24–26]. Consequently, vaccination studies present multiple optimal strategies, each optimized for a different measure. However, from a policymaker’s point of view, it is not clear 1) which criterion for the optimality of an allocation should be chosen, 2) what are the trade-offs among different measures when determining an allocation, 3) how robust is the choice of allocation to changes in assumptions, 4) how robust is the choice of allocation to a change in goals or the measures of choice, e.g., due to accumulating information on long-COVID conditions. Here, we address these issues by taking a different approach and considering the problem as one of multi-objective optimization, giving rise to a set of Pareto-optimal vaccine allocations. The Pareto-optimality approach allows to systematically evaluate the trade-offs among competing measures such as mortality and number of infections. In [23], this approach was used to evaluate, in retrospect, the performance of vaccine allocations. Here, we consider Pareto-optimality as a tool to design new vaccine allocations and to study the impact of policy decisions. Specifically, by comparing the possible outcomes of Pareto-optimal allocations (the *Pareto front*) for the scenarios in which all age-groups can be vaccinated with those obtained when young age groups are not vaccinated, we obtain a global view of the extent to which limiting eligibility for vaccination effects outcomes. More generally, such comparisons among Pareto fronts provide a way to visualize the impact of various changes in assumptions.

## 2 Methods

This section presents the mathematical model used as a basis for this study and the analytical and numerical methods used to explore questions related to optimal vaccination using the model.

### 2.1 Age-structured SIR model with vaccination

The computations in this work rely on an age-stratified SIR model [22, 27, 28]. We note that since our results concern only the herd-immunity threshold and final sizes, and since these quantities do not depend on the generation-time distribution [28], the results derived are identical to those that would be obtained from a more elaborate SEIR or a more general age-of-infection model.

The population is divided into *n* age groups. The dynamic variables are *S*_*j*_,*I*_*j*_,*R*_*j*_ and *V*_*j*_, the numbers of susceptible, infected, recovered, and vaccinated individuals in age-group *j* (1 ≤ *j* ≤ *n*).

Parameters of the model are:

- *N*_*j*_ (1 ≤ *j* ≤ *n*) is the size of age group *j*.
- *C*_*jk*_ denotes the mean number of contacts of a single member of age group *j* with members of group *k* per unit time. We denote by *C* the *n* × *n* matrix with elements $C={\left\{C_{jk}\right\}}_{j,k=1}^{n}$.
- *β*_*j*_ is the probability of infection upon contact for members of the group *j*, allowing for varying susceptibility to infection in different age groups.
- *γ* denotes the recovery rate so that $\frac{1}{\gamma }$ is the mean duration of infectivity.
- *p*_*j*_ ∈ [0, 1] is the fraction of group *j* which is vaccinated.
- *ν* is the fraction of those vaccinated for which protective immunity is generated.
- 1 − *ε* is the vaccine efficacy against infection, so that *ε* is the factor by which the probability of infection upon contact is reduced for those vaccinated.
  The case *ε* > 0, *ν* = 1 is known as ‘leaky vaccine’, and the case *ε* = 0, *ν* < 1 is known as an ‘all-or-none’ vaccine.

The dynamics is then described by the differential equations

$$\begin{array}{c} S_{j}^{\prime }\left(t\right)=-\beta_{j}S_{j}\left(t\right)\sum_{k=1}^{n}C_{jk}\cdot \frac{I_{k}\left(t\right)}{N_{k}}, \end{array}$$ (1)

$$\begin{array}{c} V_{j}^{\prime }\left(t\right)=-\varepsilon \beta_{j}V_{j}\left(t\right)\sum_{k=1}^{n}C_{jk}\cdot \frac{I_{k}\left(t\right)}{N_{k}}, \end{array}$$ (2)

$$\begin{array}{c} I_{j}^{\prime }\left(t\right)=\beta_{j}\left(S_{j}\left(t\right)+\varepsilon V_{j}\left(t\right)\right)\sum_{k=1}^{n}C_{jk}\cdot \frac{I_{k}\left(t\right)}{N_{k}}-\gamma I_{j}, \end{array}$$ (3)

$$\begin{array}{c} R_{j}^{\prime }\left(t\right)=\gamma I_{j}\left(t\right). \end{array}$$ (4)

We assume that the initial numbers of infected *I*_*j*_(0) and of recovered *R*_*j*_(0) are given. Since a proportion *p*_*j*_ of age group *j* is vaccinated, and a fraction *ν* of these will generate immunity, we have

$$\begin{array}{c} V_{j}\left(0\right)=\nu p_{j}N_{j},\;\;S_{j}\left(0\right)=N_{j}-I_{j}\left(0\right)-R_{j}\left(0\right)+\left(1-\nu \right)p_{j}N_{j}. \end{array}$$ (5)

To calculate the basic reproductive number, *R*_0_, we use the next-generation matrix

$$\begin{array}{c} M=\frac{1}{\gamma }D_{\beta }C^{T}, \end{array}$$ (6)

where *D*_*β*_ is the diagonal matrix with diagonal entries *β*_*i*_ and *C* is the country-specific contact matrix. The basic reproductive number *R*_0_ is equal to *ρ*(*M*), the spectral radius of *M* [27, 28].

### 2.2 Parameter values

Here we describe the parameter values used in the computations which were carried out.

- **Age Demographics** in all simulations were taken from the UN World Population Prospects 2019 for each country [29], using *n* = 9 age groups, of sizes *N*_*j*_ (1 ≤ *j* ≤ 9) corresponding to 10-year increments, with the last group comprising those of age 80 and older.
- **Contact matrices** $C={\left\{C_{jk}\right\}}_{j,k=1}^{n}$. We used contact matrices from [30]. Age bins in each case were originally provided in a 5-year increment, where the last age bin corresponds to ages 75 and older. We follow the procedure as in [22] to adapt the matrices into 10-year increments.
- **Susceptibility parameters** *β*_*i*_: The examples presented in this study assume the age dependent susceptibility profile for SARS-CoV-19 from [15]:
  $$\begin{array}{c} \left(\beta_{1},\cdots ,\beta_{9}\right)=\beta \cdot \left(0.4,0.38,0.79,0.86,0.8,0.82,0.88,0.74,0.74\right) \end{array}$$
  in which the relative susceptibility of age group 0–19 is roughly half of older age groups. The parameter *β* is adjusted to obtain different values of *R*_0_.
- **Vaccine efficacy** Unless otherwise specified, in what follows, we assume that the susceptibility of vaccinated individuals to infection is reduced by 90% (so *ε* = 0.1), and the risk of a vaccinated infectee to develop severe disease or to die is 50% that of a non-vaccinated infectee. By construction, this combination of parameters gives rise to an overall reduction of 95% in the risk of a vaccinated individual to develop a severe disease as estimated in controlled studies [31] and analysis of real-world data [32].
  The fraction *ν* of those vaccinated for which protective immunity is generated, is taken to be *ν* = 1.
- **Age- dependent infection fatality ratio (IFR)** We assume the age dependent IFR profile [22, 33],
  $$\begin{array}{c} \left(\eta_{1},\cdots ,\eta_{9}\right)=\left(0.00095,0.0031,0.011,0.036,0.12,0.40,1.35,4.5,15.2\right), \end{array}$$
  where, for example, *η*_9_ implies an IFR of 15.2% at ages 80 and older.
- **Initial values**: The initial values for the differential equations, which are used in the final-size formulas, are *R*_*i*_(0) = 0 for 1 ≤ *i* ≤ *n*, unless otherwise stated, that is we assume no recovered individuals. We also take *I*_*i*_(0) = 0 in the final size formula—corresponding to a negligible fraction of the population initially infected.

We note that, as far as the computations performed here are concerned, the value of parameter *γ* (recovery rate) in the model has no effect, since in the expressions for the reproductive number, as well as in the final size equations, $\frac{1}{\gamma }$ is multiplied by the parameter *β*, which is adjusted to achieve the desired value of *R*_0_. Therefore we do not need to fix a value for the parameter *γ*.

### 2.3 Computation of vaccine supply threshold

The post-vaccination effective reproduction number *R*_*v*_ is the spectral radius *ρ*(*M*_*v*_) of the next-generation matrix following vaccination

$$\begin{array}{c} M_{v}=\frac{1}{\gamma }D_{\beta }D_{\sigma }C^{T}, \end{array}$$ (7)

where *D*_*σ*_ is a diagonal matrix with diagonal entries

$$\begin{array}{c} \sigma_{j}=\frac{S_{j}\left(0\right)}{N_{j}}+\frac{V_{j}\left(0\right)}{N_{j}}\cdot \varepsilon =\frac{S_{j}\left(0\right)}{N_{j}}+\nu \cdot \varepsilon p_{j} \end{array}$$

The matrix *M*_*v*_ depends on the vaccinated fractions *p*_*j*_ in each age group, and to stress this we will denote it by *M*_*v*_(*p*_1_, ⋯, *p*_*n*_).

The vaccine supply threshold is the *minimal* vaccine coverage required for achieving herd immunity, that is attaining *R*_*v*_ = 1 [34, 35]. To compute this quantity we define, for each level 0 ≤ *p* ≤ 1 of total vaccine coverage, the minimal reproductive number attainable using allocations with total coverage *p*, that is

$$\begin{array}{c} R_{v}\left(p\right)=\underset{p_{1},p_{2},\cdots ,p_{n}}{\text{min}}\rho \left(M_{v}\left(p_{1},p_{2},\cdots ,p_{n}\right)\right) \end{array}$$

subject to the constraints

$$\begin{array}{c} 0\leq p_{j}\leq 1-\frac{R_{j}\left(0\right)}{N_{j}},\;\;\;1\leq j\leq n. \end{array}$$

$$\begin{array}{c} \sum_{j=1}^{m}p_{j}N_{j}=p\sum_{j=1}^{m}N_{j}. \end{array}$$

The minimal vaccination coverage required to achieve herd immunity is obtained by solving the equation *R*_*v*_(*p*) = 1, and the corresponding minimizer (*p*_1_, *p*_2_, ⋯, *p*_*n*_) gives the optimal vaccine allocation for achieving herd immunity.

We conduct these computations using Matlabs’ fmincon nonlinear programming solver, where the vaccine coverage *p* is gradually increased, and the initial guess used for coverage *p* + *δp* is adapted from the solution of the optimization problem for vaccine coverage *p*. We should note that in general, the optimization problem that we solve here is a non-convex one [34, 35], so we do not have theoretical guarantees that there will not exist local minima, at which the optimization algorithm could get stuck without finding the global minimum. To reduce the probability of convergence to a local minimum, we have randomized the initial point provided to the algorithm and verified that it converges to the same minimum so that we are reasonably confident that we have found the global minima.

### 2.4 Final size formula

The final-size formula [27, 28] yields the overall number of infections in each age group in terms of the model parameters, allowing us to compute the epidemic’s outcome without the need to solve the differential equations numerically.

It is convenient to reformulate the system (1)–(4) in terms of proportions

$$\begin{array}{c} s_{j}=\frac{S_{j}}{N_{j}},\;v_{j}=\frac{V_{j}}{N_{j}},\;i_{j}=\frac{I_{j}}{N_{j}},\;r_{j}=\frac{R_{j}}{N_{j}}. \end{array}$$

Obtaining

$$\begin{array}{c} s_{j}^{\prime }\left(t\right)=-\beta_{j}s_{j}\left(t\right)\sum_{k=1}^{n}C_{jk}\cdot i_{k}\left(t\right), \end{array}$$ (8)

$$\begin{array}{c} v_{j}^{\prime }\left(t\right)=-\varepsilon \beta_{j}v_{j}\left(t\right)\sum_{j=1}^{n}C_{jk}\cdot i_{k}\left(t\right), \end{array}$$ (9)

$$\begin{array}{c} i_{j}^{\prime }\left(t\right)=\beta_{j}\left(s_{j}\left(t\right)+\varepsilon v_{j}\left(t\right)\right)\sum_{k=1}^{n}C_{jk}\cdot i_{k}\left(t\right)-\gamma i_{j}\left(t\right), \end{array}$$ (10)

$$\begin{array}{c} r_{j}^{\prime }\left(t\right)=\gamma i_{j}\left(t\right). \end{array}$$ (11)

Assuming that the initial fractions of infected *i*_*j*_(0) and of recovered *r*_*j*_(0) are given, (5) translates into

$$\begin{array}{c} v_{j}\left(0\right)=\nu p_{j},\;\;s_{j}\left(0\right)=1-i_{j}\left(0\right)-r_{j}\left(0\right)+\left(1-\nu \right)p_{j}. \end{array}$$

From (8) and (9) we have

$$\begin{array}{c} \frac{s_{j}^{\prime }\left(t\right)}{s_{j}\left(t\right)}=-\beta_{j}\sum_{k=1}^{n}C_{jk}\cdot i_{k}\left(t\right),\;\frac{v_{j}^{\prime }\left(t\right)}{v_{j}\left(t\right)}=-\varepsilon \beta_{j}\sum_{k=1}^{n}C_{jk}\cdot i_{k}\left(t\right), \end{array}$$

which upon integration yields

$$\begin{array}{c} \text{log}\frac{s_{j}\left(\infty \right)}{s_{j}\left(0\right)}=-\beta_{j}\sum_{k=1}^{n}C_{jk}\int_{0}^{\infty }i_{k}\left(s\right)ds,\;\text{log}\frac{v_{j}\left(\infty \right)}{v_{j}\left(0\right)}=-\varepsilon \beta_{j}\sum_{k=1}^{n}C_{jk}\int_{0}^{\infty }i_{k}\left(s\right)ds, \end{array}$$

or

$$\begin{array}{c} \begin{array}{cc} s_{j}\left(\infty \right) & =s_{j}\left(0\right)\;\text{exp}\;[-\beta_{j}\sum_{k=1}^{n}C_{jk}\int_{0}^{\infty }i_{k}\left(s\right)ds],\\ v_{j}\left(\infty \right) & =v_{j}\left(0\right)\;\text{exp}\;[-\varepsilon \beta_{j}\sum_{k=1}^{n}C_{jk}\int_{0}^{\infty }i_{k}\left(s\right)ds]. \end{array} \end{array}$$ (12)

Note that, assuming *s*_*i*_(0) ≠ 0, (12) implies the relation

$$\begin{array}{c} v_{i}\left(\infty \right)=v_{i}\left(0\right){\left(\frac{s_{i}\left(\infty \right)}{s_{i}\left(0\right)}\right)}^{\varepsilon }. \end{array}$$

Summing (8)–(10), we have

$$\begin{array}{c} i_{j}^{\prime }\left(t\right)=-s_{j}^{\prime }\left(t\right)-v_{j}^{\prime }\left(t\right)-\gamma i_{j}\left(t\right), \end{array}$$

which, upon integration, gives

ij(∞)−ij(0)=sj(0)−sj(∞)+vj(0)−vj(∞)−γ∫0∞ij(t),

or

$$\begin{array}{c} \int_{0}^{\infty }i_{j}\left(t\right)=\frac{1}{\gamma }[s_{j}\left(0\right)-s_{j}\left(\infty \right)+v_{j}\left(0\right)-v_{j}\left(\infty \right)+i_{j}\left(0\right)]. \end{array}$$ (13)

Since *s*_*j*_(*t*) + *v*_*j*_(*t*) + *i*_*j*_(*t*) + *r*_*j*_(*t*) = 1 for all *t*, we have

$$\begin{array}{c} s_{j}\left(0\right)+v_{j}\left(0\right)+i_{j}\left(0\right)=1-r_{j}\left(0\right),\;s_{j}\left(\infty \right)+v_{j}\left(\infty \right)=1-r_{j}\left(\infty \right), \end{array}$$ (14)

and can write (13) as

$$\begin{array}{c} \int_{0}^{\infty }i_{j}\left(t\right)=\frac{1}{\gamma }[r_{j}\left(\infty \right)-r_{j}\left(0\right)], \end{array}$$

so that (12) yields

$$\begin{array}{ccc} s_{j}\left(\infty \right) & =s_{j}\left(0\right)\;\text{exp}\;[-\frac{\beta_{j}}{\gamma }\sum_{k=1}^{n}C_{jk}[r_{k}\left(\infty \right)-r_{k}\left(0\right)]], & \left(15\text{a}\right)\\ v_{j}\left(\infty \right) & =v_{j}\left(0\right)\;\text{exp}\;[-\varepsilon \frac{\beta_{j}}{\gamma }\sum_{k=1}^{n}C_{jk}[r_{k}\left(\infty \right)-r_{k}\left(0\right)]]. & \left(15\text{b}\right) \end{array}$$

Combining (14) and (15) yields

$$\begin{array}{c} \begin{array}{cc} 1-r_{j}\left(\infty \right)= & s_{j}\left(0\right)\;\text{exp}\;[-\frac{\beta_{j}}{\gamma }\sum_{k=1}^{n}C_{jk}[r_{k}\left(\infty \right)-r_{k}\left(0\right)]]+\\ & v_{j}\left(0\right)\;\text{exp}\;[-\varepsilon \frac{\beta_{j}}{\gamma }\sum_{k=1}^{n}C_{jk}[r_{k}\left(\infty \right)-r_{k}\left(0\right)]], \end{array} \end{array}$$

or, defining *z*_*j*_ = *r*_*j*_(∞) − *r*_*j*_(0) to be fraction of group *j* infected throughout the post-vaccination period:

$$\begin{array}{c} 1-r_{j}\left(0\right)-z_{j}=s_{j}\left(0\right)\;\text{exp}\;[-\frac{\beta_{j}}{\gamma }\sum_{k=1}^{n}C_{jk}z_{k}]+v_{j}\left(0\right)\;\text{exp}\;[-\varepsilon \frac{\beta_{j}}{\gamma }\sum_{k=1}^{n}C_{jk}z_{k}],\;\;\;1\leq j\leq n. \end{array}$$

Numerically solving this system of equations yields the fractions *z*_*j*_.

**Fig 1:**
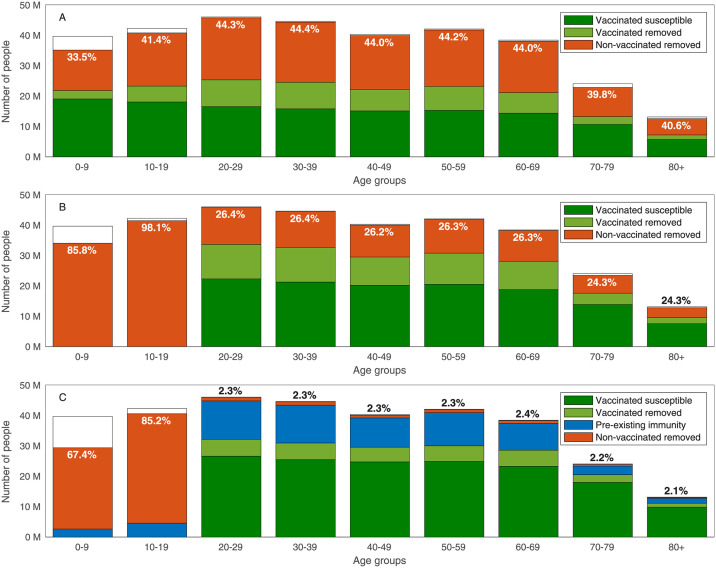
Final size of the epidemic. Final size of an epidemic spreading with basic reproduction number of *R*_0_ = 6 after 55% of the population of the USA is vaccinated with no age prioritization. Removed population refers to those recovered or dead. The computation considers a vaccination campaign in which A: Vaccines are allocated to all ages. B: Vaccine allocation is limited to ages 20 and above. C: Vaccine allocation is limited to ages 20 and above, 20% of the population has preexisting immunity recovered from COVID-19, and the prevalence of active cases is 0.5% of the population. The text in all graphs corresponds to the percent of non-vaccinated removed individuals in each age group.

For example, we compute the final size of an epidemic spreading after 55% of the population of the USA is vaccinated with no age prioritization, i.e., 55% of each age group is vaccinated. Considering a post-COVID basic reproduction number of *R*_0_ = 6, we observe that by the end of the epidemic, 40.6% of the population above the age of 80 will be infected without the protection of a vaccine, see Fig 1A. When vaccines are homogeneously allocated only to adults (ages 20 and over), the portion of the population above the age of 80 that is infected without the vaccine’s protection drops to 24.3%. In this case, however, the portion of children in the age group of 10–19 that are infected increases to 98.1%, see Fig 1B. The above examples assume that the entire population is either susceptible or vaccinated at the end of the vaccination campaign. To model in a more realistic manner, we allow for preexisting immunity due to recovery, as well as for the prevalence of active cases, see Fig 1C.

### 2.5 Computation of optimal vaccine allocations

For a given vaccine allocation ${\left\{p_{i}\right\}}_{i=1}^{n}$ where *p*_*i*_ is the fraction of age group *i* which is vaccinated, we use the final size formula to compute the outcomes in terms of the fraction of each age group infected *z*_*j*_ (1 ≤ *j* ≤ *n*). The function *f*(*p*_1_, *p*_2_, ⋯, *p*_*n*_) to be minimized in the case that the aim is to minimize the number of infections is

$$\begin{array}{c} f_{I}\left(p_{1},p_{2},\cdots ,p_{n}\right)=\sum_{j=1}^{n}z_{j}\cdot N_{j}, \end{array}$$

while if the aim is minimizing mortality we take

$$\begin{array}{c} f_{M}\left(p_{1},p_{2},\cdots ,p_{n}\right)=\sum_{j=1}^{n}\eta_{j}\cdot z_{j}\cdot N_{j}, \end{array}$$

where *η*_*j*_ (1 ≤ *j* ≤ *n*) are the infection fatality rates (IFR) in each age group.

Given the total fraction *p* of the population to be vaccinated, we consider the following optimization problem (with *f* = *f*_*I*_, *f* = *f*_*M*_ or a convex combination of these functions):

$$\begin{array}{c} \text{min}\;f(p_{1},p_{2},\cdots ,p_{n}) \end{array}$$

subject to

$$\begin{array}{c} \sum_{i=1}^{n}p_{i}N_{i}=p\sum_{i=1}^{n}N_{i}\;(\text{Total}\;\text{vaccine}\;\text{allocation}\;\text{is}\;\text{equal}\;\text{to}\;\text{vaccine}\;\text{supply}), \end{array}$$

$$\begin{array}{c} 0\leq p_{j}\leq 1-r_{j}\left(0\right)\;(\text{Vaccine}\;\text{allocation}\;\text{only}\;\text{to}\;\text{susceptible}\;\text{individuals}). \end{array}$$

This optimization problem is solved using Matlabs’ fmincon nonlinear programming solver.

The inequality constraints can readily be modified so that vaccine allocation also does not exceed a given portion *α*_*j*_ of age group *j*,

$$\begin{array}{c} 0\leq p_{j}\leq \text{min}\left(\alpha_{j},1-r_{j}\left(0\right)\right). \end{array}$$

This modification enables to account for age groups for which vaccination is not approved (by setting *α*_*j*_ = 0), as well as for vaccine hesitancy, logistical difficulties in reaching the entire population of an age group, or a portion of the population who cannot be vaccinated due to medical conditions or allergies.

### 2.6 The Pareto front and its computation

The Pareto front is a tool that allows us to examine the trade-offs among competing measures for the effectiveness of a vaccination campaign—in our case, the trade-off between minimizing the number of infections (attack rate) and mortality. For a given vaccination coverage, an outcome (*Z*, *M*) (attack rate and mortality) is called *feasible* if it can be achieved by a suitable allocation of vaccines satisfying the coverage constraint. An outcome is called *Pareto optimal* if it is feasible, and if there do not exist feasible outcomes (*Z*′, *M*′) which improve upon it both in terms of attack rate and in terms of mortality (*Z*′ < *Z*, *M*′ < *M*). The set of Pareto-optimal outcomes is called the *Pareto front*. We aim to compute the Pareto front and display it graphically in the plane of outcomes (*Z*, *M*).

Each point on the Pareto front corresponds to the mortality minimizing vaccine allocation with a given number of infections. To compute the Pareto front, we first compute its endpoints—namely, we compute the vaccine allocation minimizing attack rate and the vaccine allocation minimizing mortality, with corresponding outcomes (*Z*_0_, *M*_0_) and (*Z*_*L*_, *M*_*L*_), respectively. We conduct these computations using Matlabs’ fmincon nonlinear programming solver. To avoid convergence of the optimization algorithm to a local minimum, we run the solver with a set of random initial guesses.

The computation of the endpoints of the Pareto front determines the range for the attack rate along the Pareto front and allows to determine a grid of *L* points

$$\begin{array}{c} Z_{l}=Z_{0}+\frac{Z_{L}-Z_{0}}{L}\cdot l,\;l=1,\cdots ,L, \end{array}$$

along which the Pareto front is sampled. The optimal allocations along the Pareto front are computed sequentially from one end of the Pareto front to the other by solving the constrained optimization problem of finding the mortality minimizing vaccine allocation with a given number *Z*_*l*_ of infections for *l* = 1, 2, ⋯, *L* − 1:

$$\begin{array}{c} \text{min}\;f_{M}\left(p_{1},\cdots ,p_{n}\right), \end{array}$$

subject to

$$\begin{array}{c} f_{I}\left(p_{1},\cdots ,p_{n}\right)=Z_{l} \end{array}$$

$$\begin{array}{c} \sum_{i=1}^{n}p_{i}N_{i}=p\sum_{i=1}^{n}N_{i}, \end{array}$$

$$\begin{array}{c} 0\leq p_{j}\leq 1-r_{j}\left(0\right). \end{array}$$

The initial guesses used for each optimization problem at stage *l* is a set of random allocations around the optimal allocation found for the point *l* − 1.

In some cases, we have observed that the direction of sweep from one end to the other affects the results obtained. To eliminate this factor, we sweep in the opposite direction and, if needed, update the optimal allocation computed. Namely, we recompute the Pareto front at points *Z*_*l*_ for *l* = *L* − 1, *L* − 2, ⋯, 1 where the initial guess for each optimization problem is the optimal allocation found for the point *l* + 1.

## 3 Results

In what follows, we consider scenarios of a partial return to normality, to a basic reproduction number of *R*_0_, after vaccination efforts are completed. We illustrate our results using parameters corresponding to the USA demography and contact structure and later consider how the different demographic structures of other countries affect the results. We note that *R*_0_ is the reproductive number in the absence of vaccination and preexisting immunity due to recovery.

The goal of the first examples is to illustrate the methodology. Accordingly, these examples do not necessarily reflect up-to-date vaccine coverage and are simplistic in the sense that they do not account for preexisting immunity, hesitance, and other factors of relevance. These illustrative examples are followed by a real-would example of the reevaluation of the vaccination campaign in Israel in October 2021. This example includes various factors of relevance such as preexisting immunity. Furthermore, a systematic study of the effect of changes in various parameters such as hesitancy or vaccine coverage is provided in the supplementary material, see S1 Text.

### 3.1 Vaccination coverage required for herd immunity

Achieving herd immunity requires the vaccination of a sufficiently large sub-population. The required coverage can be minimized by allocating the vaccines to different age groups in an optimal manner. Here, for each value of *R*_0_, we compute the minimal vaccine coverage *V*_threshold_ necessary to reach herd immunity and the corresponding vaccine allocation among the eligible age groups that achieves this goal. See Section 2.3 for details on these computations.

**Fig 2:**
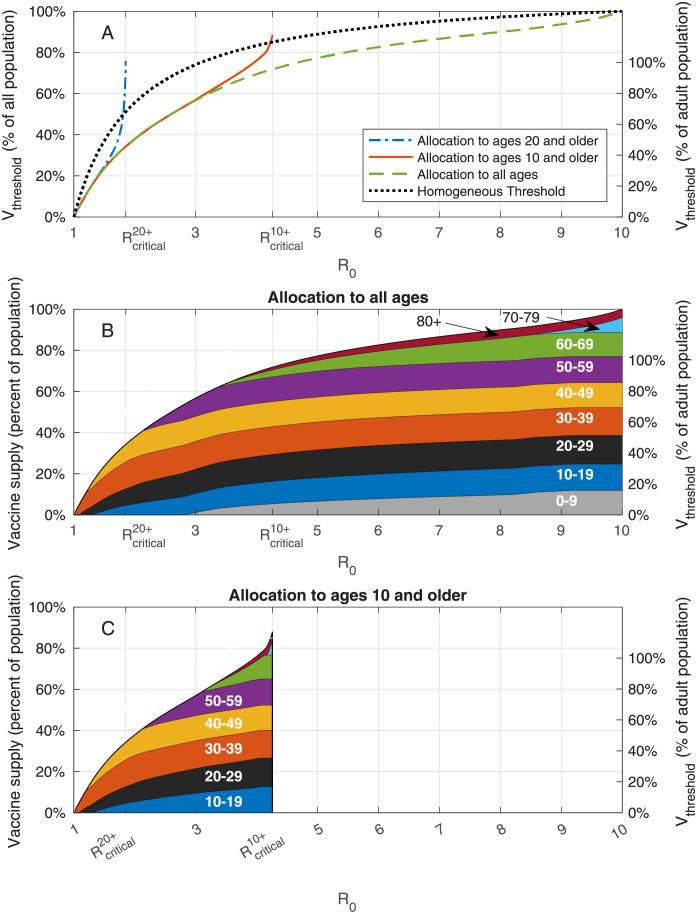
Vaccination coverage required for herd immunity. A: Vaccine coverage *V*_threshold_ required to achieve herd immunity threshold as a function of the reproduction number *R*_0_ for the USA demography and contact structure where $R_{\text{critical}}^{20+}$ and $R_{\text{critical}}^{10+}$ correspond to beproduction numbers at which herd immunity cannot be achieved without vaccination of age groups 0 − 19 and 0 − 9, respectively. B: Vaccine allocations at which herd immunity is achieved at minimal vaccine coverage and when all the population is eligible for vaccination. C: Same as B, but when ages ten and older are eligible for vaccination.

We first examine the case in which the entire population is eligible for vaccination. The results vary with the reproductive number *R*_0_. For example, when *R*_0_ is less than ≈ 1.1, the required vaccine supply is relatively small and is allocated solely to age group 30–39, see Fig 2B. Then, as *R*_0_ increases, the required vaccine supply *V*_threshold_ gradually increases, and its allocation is extended to other age groups. The optimal vaccine allocations are not necessarily allocations to those who do the most transmitting. Indeed, as *R*_0_ increases, new age groups are typically added to the allocation before the coverage of the age groups already present in the allocation has reached 100%. For *R*_0_ = 3, herd immunity can be achieved by vaccinating roughly 56% of the population in an optimal way, see Fig 2A. In comparison, if vaccines are allocated *pro rata* (in proportion to the size of age groups), achieving herd immunity requires vaccination of 75% of the population, taking into account 90% vaccine efficacy against infection.

To assess the population-level impact of the vaccination of children younger than the age of ten on the prospects for achieving herd immunity, we repeat the analysis while restricting the allocation of vaccines to ages ten and older, and compute the corresponding threshold curve $V_{\text{threshold}}^{10+}$ as a function of *R*_0_. We observe that for low values of *R*_0_ the threshold curve $V_{\text{threshold}}^{10+}$ coincides with the threshold curve *V*_threshold_ corresponding to the case in which all ages are vaccine eligible, see Fig 2B in comparison with Fig 2C. The implication is that for values of *R*_0_ < 3.1, any allocation achieving herd immunity and including age group 0 − 9 would be *suboptimal* in the sense that its vaccine coverage is larger than the minimum required. For *R*_0_ > 3.1, the two curves diverge. The divergence stems from the fact that, for these values of *R*_0_, all age groups (including children 0 − 9) partake in the optimal allocation (see Fig 2B). Hence, for these values of *R*_0_, any allocation achieving herd immunity and *not* including age group 0 − 9 would be *suboptimal* in that it would require a higher level of vaccination overall than the minimal achievable coverage. As *R*_0_ increases further, the threshold curve $V_{\text{threshold}}^{10+}$ rapidly increases up to its maximal value, which corresponds to 100% of the eligible population at $R_{0}=R_{\text{critical}}^{10+}\approx 4.25$. When $R_{0}>R_{\text{critical}}^{10+}$, reaching herd immunity becomes *impossible* if children under the age of 10 are not vaccinated.

In case vaccines are not allocated to age group 0–19, we find that herd immunity is achievable only for rather low reproductive numbers, $R_{0}<R_{\text{critical}}^{20+}\approx 1.85$. For higher values of *R*_0_, the spread of the infection is sustained solely by the population under the age of 20. Therefore, for typical values of *R*_0_ of SARS-CoV-19 and its variants, vaccination of age group 10–19 is essential for achieving herd immunity.

**Fig 3:**
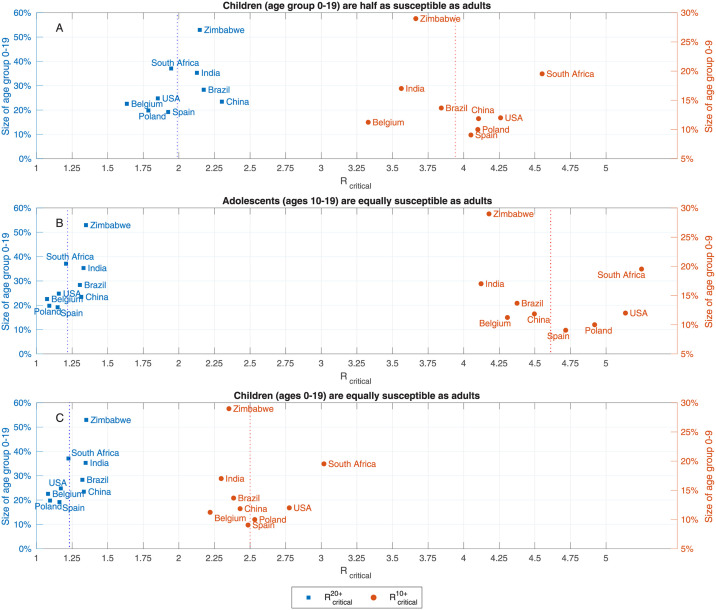
Critical reproduction numbers. Reproduction numbers $R_{\text{critical}}^{20+}$ and $R_{\text{critical}}^{10+}$ at which herd immunity cannot be achieved without vaccination of age groups 0 − 19 and 0 − 9, respectively. Computed using A: age-dependent susceptibility profile of SARS-CoV-19: Ages 0–19 are roughly half as susceptible as adults [15]. B: Same susceptibility profile as in A, but with increased susceptibility in the age group 10–19: Ages 10–19 are as susceptible as adults. C: Same susceptibility profile as in A, where ages 10–19 are as susceptible as adults.

The above example, presented in Fig 2, relies on the demographic structure and the contact matrix of the USA [30]. We have also examined the critical reproduction numbers $R_{\text{critical}}^{10+}$ and $R_{\text{critical}}^{20+}$ for eight additional countries using the contact matrices estimated in [30], and found the results to be similar, see Fig 3A. Namely, we found that despite the diversity of the countries examined, $1.6<R_{\text{critical}}^{20+}<2.3$, and $3.3<R_{\text{critical}}^{10+}<4.6$. In particular, we observe that whether vaccinating children is necessary for reaching herd immunity is not determined by looking at the percentage of children in the population, as a naive calculation based on a homogeneous-population model would imply. For example, in the case of Zimbabwe, with 53% of its population in age group 0–19, we computed $R_{\text{critical}}^{20+}\approx 2.15$, which is higher than $R_{\text{critical}}^{20+}\approx 1.8$ computed for Poland for which the size of age group 0–19 is 20% of the total population. Instead, the critical factor is the level of assortativity of mixing within the children sub-population, as reflected in the contact matrix.

An important consideration in undertaking a policy, such as aiming to achieve herd immunity through vaccination, is its robustness to uncertainties or possible changes in conditions. For example, if *R*_0_ varies as a result of a new viral variant, herd immunity may be lost, leading to an epidemic surge. Since the allocations aiming to achieve herd immunity focus on younger population that do the most transmitting, they leave the high-risk population exposed in case of an epidemic spread. For example, if herd immunity is achieved for *R*_0_ < 2.9 by vaccinating 55% of the population, and *R*_0_ increases to *R*_0_ = 4 or *R*_0_ = 6, the resulting epidemic surge would result in 2.26*M* or 3.3*M* fatalities, respectively. In comparison, as will be shown subsequently, other allocations with the same coverage can achieve 0.26*M* or 0.45*M* fatalities, respectively. We thus conclude that aiming for herd immunity is not a robust strategy.

### 3.2 Optimal allocations for minimizing mortality

We now examine cases in which the vaccination campaign is followed by an epidemic outbreak since herd immunity is not achieved with given coverage at the range of *R*_0_ which the policy is designed for.

In what follows, we assess the outcomes of post-vaccination epidemic spread by considering two widely employed measures: attack rate (overall number of infections) and mortality, see Section 2.4 for details. The example below considers a vaccine coverage of 55% of the population, when either all ages, only ages 10 and older or only ages 20 and older are eligible for vaccination. In each case, we assume *R*_0_ is above the threshold at which herd immunity is achievable with the given vaccine coverage.

**Fig 4:**
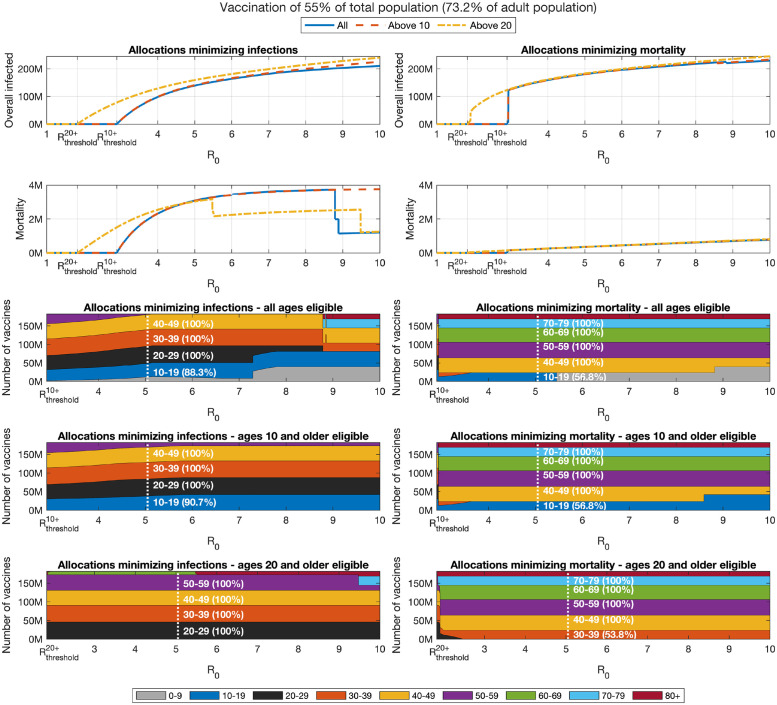
Impact of change in reproduction number. Overall infections of non-vaccinated individuals (top graphs) and overall mortality (centered graphs) as function of the basic reproduction number *R*_0_ after completion of a vaccination campaign for allocations minimizing infections (left panels) and allocations minimizing mortality (right panels). The outcomes are presented for the following cases: All ages are eligible for vaccination (solid), only ages 10 and older are eligible (dashes), and only ages 20 and older are eligible (dash-dots). Bottom panels present the corresponding allocations.

Let us first consider the case of vaccine allocations aimed at minimizing mortality, see Section 2.5 for details on the computation of such optimal allocations. In this case, as expected, as herd immunity is lost at *R*_0_ > *R*_threshold_, we observe that optimal allocations for minimizing mortality include the older age groups—see the bottom right panels of Fig 4. These allocations are associated with a significant number of infections—see the top right panel. The optimal allocation varies only slightly with *R*_0_. At high *R*_0_, we observe children are prioritized. This phenomenon will be discussed subsequently.

### 3.3 Minimizing infections

We now consider allocations minimizing the number of infections, see the left panels of Fig 4. For the lower range of values of *R*_0_ (*R*_0_ < 7), the structure of the allocations optimized to minimize infections, displayed in the bottom left panels, varies only slightly with *R*_0_. Particularly, in this range of the basic reproduction number, as *R*_0_ increases, the allocation prioritizes younger age groups, so that when of *R*_0_ > 4 a small fraction of age group 0–9 is included in the allocation. However, at higher values of *R*_0_ (*R*_0_ > 7), we observe sudden and counter-intuitive shifts in the optimal allocation. The first such transition occurs at *R*_0_ ≈ 7.3, at which the optimal allocation to age group 0–9 abruptly increases, at the expense of the 20–29 age group. A second transition occurs at *R*_0_ ≈ 8.8, at which the allocation of vaccines to those of age 70 and older is increased at the expense of vaccine allocations to well-connected age groups such as 20–39—this transition also incidentally leads to a drop in mortality due to the larger protection which the older age groups receive. This shifting of vaccine resources to the less connected groups seems quite surprising. Intuitively, an explanation is that at sufficiently high values of *R*_0_, in view of the fact that the vaccine provides imperfect protection, vaccines breakthroughs in those groups who have more contacts become so common that it becomes inefficient to allocate vaccines to those groups when the aim is to minimize infections. Instead, the optimal vaccine allocation is to withdraw to the second line of defense and vaccinate age groups that are the least likely to be infected, either due to being less connected (older age groups) or to being less susceptible to infection (children under 10). A comprehensive study of this phenomenon is presented in [36].

### 3.4 Pareto-optimal allocation of vaccines

Sections 3.2–3.3 considered vaccine allocations that minimize one of two basic measures: attack rate (overall number of infections), and mortality. Due to the age-dependent infection fatality ratio there is a trade-off between infections and mortality. The results presented in Fig 4 show that the trade-offs between infections and mortality are considerable. For example, when *R*_0_ = 4 and vaccines can be allocated to all ages, mortality ranges from 0.25M to 2.35M, while overall infections range from 100M to 162M, for vaccine allocations aimed at minimizing number of infections, or mortality, respectively. In what follows, we utilize a multi-criteria Pareto optimality approach to systematically evaluate the trade-offs involved among the two measures.

**Fig 5:**
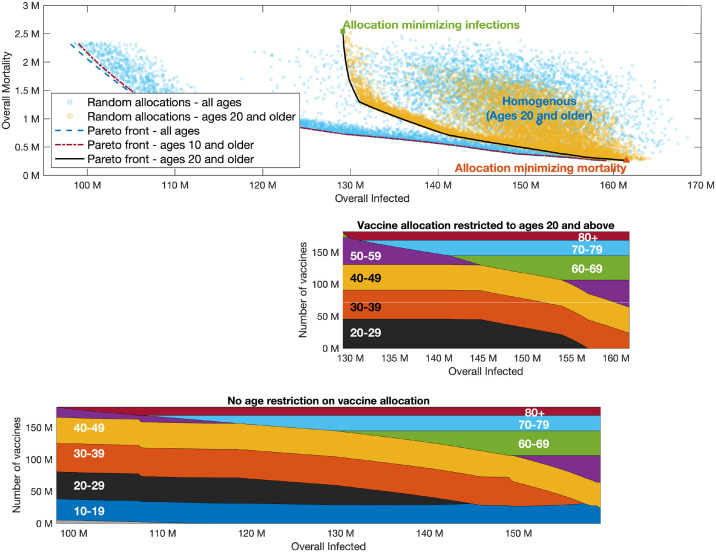
Pareto front. *R*_0_ = 4. Top graph presented outcomes of random allocations when all ages are eligible for vaccination (square blue markers) and only ages 20 and older are eligible (round yellow markers). Super-imposed are the Pareto fronts in the case ages 20 and older are eligible for vaccination (black solid), ages 10 and older are eligible (yellow dash-dotted) and when all ages are eligible (purple dashed). The latter two curves are indistinguishable. Bottom graphs present vaccine allocation along the Pareto fronts in the case of ages 20 and older are eligible for vaccination, and when all ages or ages 10 and older are eligible for vaccination.

**Fig 6:**
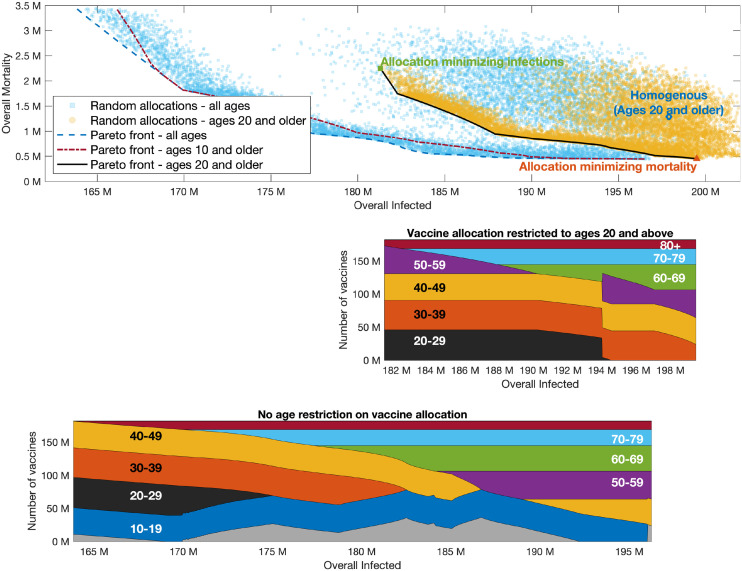
Pareto front. Same as Fig 5 with *R*_0_ = 6.

**Fig 7:**
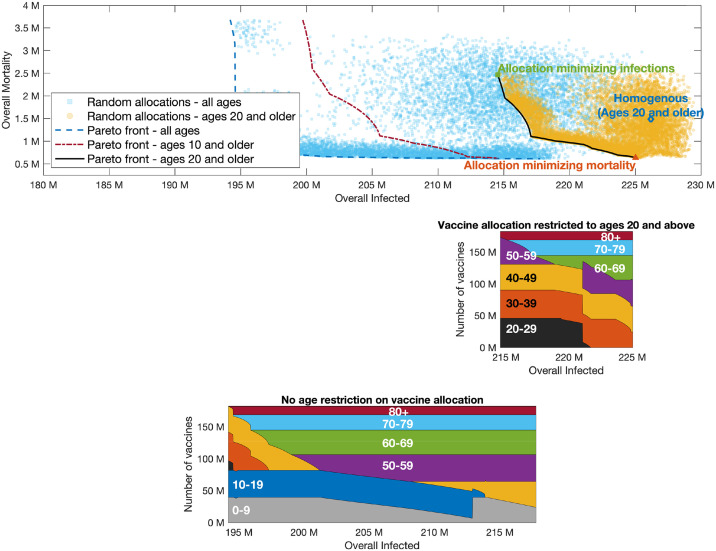
Pareto front. Same as Fig 5 with *R*_0_ = 8.

It is instructive to first consider a wide set of randomly chosen vaccine allocations, not necessarily designed to be optimal in any sense. For each allocation, we plot the possible outcomes in a plane so that the coordinates of a point correspond to the outcome of a given vaccine allocation in terms of infections and mortality. The yellow points presented in Fig 5A present the outcomes corresponding to allocations with a coverage of 55% of the US population, but restricted to adults of age 20 and over (providing coverage of 73.2% of this group), assuming *R*_0_ = 4 in the post-vaccination period. The point highlighted with a diamond marker corresponds to a *pro rata* allocation with no prioritization among those aged 20 and over, giving rise to mortality of roughly 750,000 individuals and an overall number of ∼150 million infected individuals. Inspection of the random allocations shows that many alternative allocations achieve better outcomes in both senses, namely reduce both infections and mortality compared to the *pro rata* allocation. Therefore, we consider the curve in the plane of possible outcomes (infections, mortality) which represents the *Pareto front*, the set of outcomes that cannot be improved upon in both senses by changing the allocation, see, e.g., the black solid curve in Fig 5. The choice among outcomes on the Pareto front (and the corresponding vaccine allocations) depends on one’s weighing of the importance of the two measures. The vaccine allocations corresponding to outcomes along the Pareto front are presented in the bottom panel of Fig 5. The right endpoint of the Pareto front represents the outcome corresponding to an allocation chosen so that mortality is minimized, while the left endpoint represents the outcome of the allocation minimizing infections. As expected, the allocation minimizing infections can be seen to prioritize younger age groups, while the allocation minimizing mortality includes older age groups. Moving along the Pareto front, we observe a rather complicated structure of variation in the allocations. For example, we see that the 40–49 age group is included in both the allocation minimizing infections and the allocation minimizing mortality, but it is not included in an intermediate range along the Pareto front. Analogous results for higher reproductive numbers *R*_0_ = 6, 8 are given in Figs 6 and 7, respectively.

Examining the role of children’s vaccination, we observe that age group 10–19 is included in the optimal allocation along the entire Pareto front. This implies that restricting vaccination to adults over 20 will worsen outcomes. The extent to which outcomes are degraded by restricting eligibility to those over 20 can be gauged by comparing the two Pareto fronts in the top part of Fig 4. Thus, for example, when *R*_0_ = 4, if vaccines are restricted to the 20+ age group, then the minimal number of infections that can be achieved is 129 million, and the allocation achieving this outcome would given rise to a mortality of 2.5 million. If those of age 10–19 become eligible, and an allocation generating the same number of infections is chosen, mortality is reduced by 70% to 0.72 million. Similarly, for higher values of *R*_0_, the outcomes on the Pareto front corresponding to vaccination of all age groups show significant improvement over the outcomes on the Pareto front when restricting vaccination to ages 20+.

On the other hand, the significance of the effect of restricting vaccination to adolescents over the age of 10 depends on the basic reproductive number *R*_0_. When *R*_0_ = 4, the allocations along the entire Pareto front either include only a very small fraction of the 0–9 age group or do not include it at all, so that the Pareto fronts corresponding to the two cases are nearly indistinguishable. When *R*_0_ = 6 (Fig 6), the allocations along the Pareto front do include a large fraction of the 0–9 age group, but the differences between the outcomes on the Pareto front when all ages are eligible, compared to those in which only ages 10 and older are eligible, are quite small. However, when *R*_0_ = 8 (Fig 7), we observe a significant difference between the Pareto fronts in these two cases, especially in the part of the Pareto front giving more emphasis to preventing infections.

### 3.5 Sensitivity to changes in assumptions

Our baseline examples, presented in Figs 2, 3A and 4–7 consider a specific set of assumptions concerning, e.g,. vaccine efficacy and vaccine hesitancy. Of particular significance in examining the role of childrens’ vaccinations, these computations all adopt the age dependent susceptibility profile estimated in [15], in which the relative susceptibility of age group 0–19 is roughly half that of older age groups. We now briefly consider the impact of changes in these assumptions on the optimal allocation. See S1 Text for additional details.

**Fig 8:**
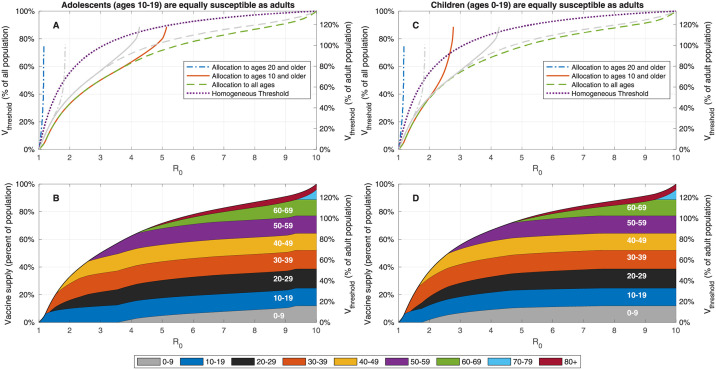
Effect of change in childrens’ susceptibility on vaccination coverage required for herd immunity. A,C: Vaccine coverage *V*_threshold_ required to achieve herd immunity threshold as a function of the reproduction number *R*_0_ for the USA demography and contact structure. The gray curves correspond to the case in which the relative susceptibility of age group 0–19 is half that of adults. B,D: Vaccine allocations at which herd immunity is achieved at minimal vaccine coverage and when there is no age restriction on vaccine allocation.

We first study the impact of changes in the age-dependent susceptibility by considering a modified age-dependent susceptibility profile in which adolescents (age group 10–19) are equally susceptible as adults, and an additional modified profile in which members of age group 0–19 are equally susceptible as adults. Fig 8 presents the vaccination coverage required for herd immunity and the vaccine allocations that lead to herd immunity at minimal vaccination coverage under the two alternative assumptions on children’s susceptibility to infection. As expected, when the susceptibility of adolescents is higher than the susceptibility of younger children, the allocations designed to achieve herd immunity at minimal coverage dedicate larger portions of vaccines to age group 10–19 at the expense of vaccine allocation to age group 0–9. Herd immunity can be achieved without vaccinating children of ages 0–9 for *R*_0_ up to 4.2.

If we assume that all members of age group 0–19 are equally susceptible as adults, vaccination of age group 10–19, and to a lesser extent, vaccination of age group 0–9 becomes of higher priority—indeed in this case herd immunity cannot be reached without vaccinating children in age group 0–9 for *R*_0_ > 2.6. Fig 3B and 3C show similar behavior when the demographic structure and contact matrices for other countries are used.

**Fig 9:**
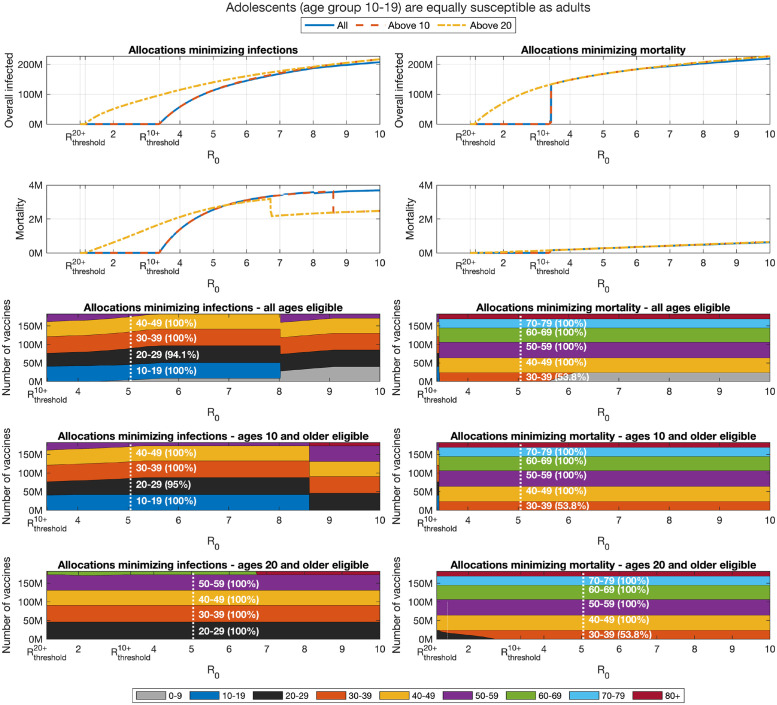
Impact of change in reproduction number when adolescents (age group 10–19) are equally susceptible as adults. Same as Fig 5 except the susceptibility of age group 10–19 is increased by factor of 2 to that of older age groups. Susceptibility of age group 0–9 is not modified.

**Fig 10:**
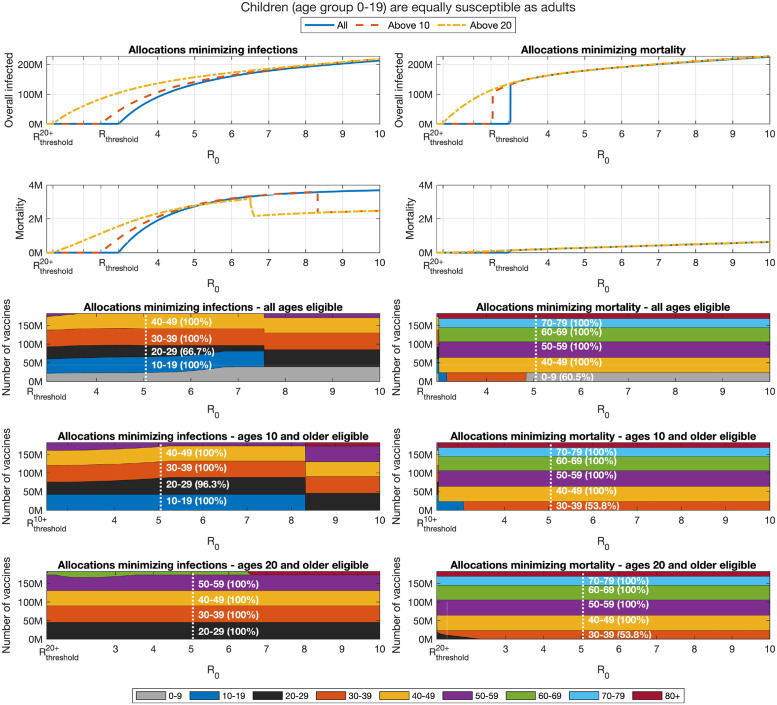
Impact of change in reproduction number when children (age group 0–19) are equally susceptible as adults. Same as Fig 5 except the susceptibility of age group 0–19 is increases by factor of 2 to that of older age groups.

Examining the outcomes associated with optimal vaccination when herd immunity cannot be achieved, we find that, as expected, when the susceptibility of children is higher, and at moderate values of *R*_0_, the allocations minimizing infections dedicate larger portions of vaccines to the age group 0–19, see Figs 9 and 10. Nevertheless, the allocations minimizing mortality do not change. However, for sufficiently large values of *R*_0_, the increased susceptibility of children has an opposite effect and leads to reduced priority given to their vaccination.

To test the impact of changes in the contact matrix on optimal allocations we modify the USA contact matrix by randomly perturbing its elements by 0–10%. The main effects of such noise are shifting of the ends of the Pareto fronts, and shifting of transitions point where vaccine allocations shifts from one group to another. These changes are comparable to the level of noise, e.g., in the example provided, 10% perturbation leads to as much as 7% shift in the end of the Pareto front. This demonstrates the utility of considering the entire Pareto front as a measure for the robustness of the allocations. See Section S1.1 in S1 Text for additional details.

Finally, we consider the effect of changes in other assumptions concerning, e.g,. vaccine efficacy or vaccine hesitancy, see S1 Text for additional details. In particular, as expected, increase of vaccine coverage allows allocation of vaccines to age groups that are otherwise at lower priority for vaccination. Similarly, preexisting immunity in age groups prioritized for vaccination allows diverting the allocation of vaccines to other age groups that are otherwise at lower priority for vaccination. Vaccine hesitancy, modelled as a constraint on the maximal fraction of each age-group that can be vaccinated, also leads to reallocation of vaccines from age groups prioritized for vaccination to those of lower priority.

## 4 Application: Israel as a case study

In the above, we focused on the case in which limited availability of vaccines implies a need to consider optimized allocations. In what follows, we consider a real-world example from Israel which demonstrates how the methodology presented in this work applies more broadly, also to cases in which a large fraction of the population has already been vaccinated, and in which vaccine shortage is not a key limiting factor. Particularly, we account for the large portion of the Israeli population already vaccinated or recovered, and ask: if it is possible to vaccinate an additional small portion of the population—how should the vaccines be distributed among the different age groups to achieve optimal epidemic outcomes going forward? Understanding whom it is most beneficial to vaccinate reveals the weak spots of vaccination coverage in Israel and can help direct efforts in raising public awareness, as well as contribute to the discussions on extending vaccine eligibility to younger age groups.

Relevant data, updated to October 2021, is acquired from the Israeli Ministry of Health, and parameters such as contact matrices and age-dependent mortality rates are customized to Israel. An account of the statistical analysis and calibration procedures used to extract these parameters will be presented elsewhere.

As of October 2021, 57% of the Israeli population had been fully vaccinated, i.e., had received a booster dose or a second dose within the preceding 6 months. Vaccine efficacy of 90% against infection is assumed for these individuals. An additional 9.5% had received their second dose of vaccine over 6 months previously, and are considered as non-vaccinated in the computation. Additionally, 13.8% of the population is known to be recovered.

**Fig 11:**
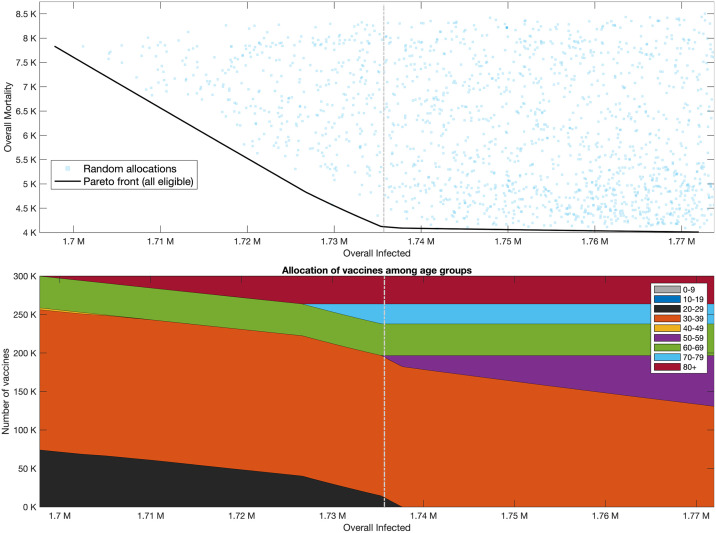
Pareto front—Vaccination of an additional 0.3 million people in Israel. Top graph presents outcomes of random allocations when all ages are eligible for vaccination (square blue markers). Black solid curve is the Pareto front when all ages are eligible for vaccination. Bottom Graphs: Vaccine allocations along the Pareto front.

We now consider the optimal allocation of additional vaccines, namely: if an additional supply of 0.3 million vaccines can be administered, we ask how they should be allocated to different age groups. To answer this question, we compute the Pareto front of outcomes following the administration of the additional supply of vaccines, when *R*_0_ = 6. Based on the data concerning second dose uptake in Israel, we assume a maximal vaccine coverage of 93% per age group. We find that additional mortality changes considerably along the Pareto front, see Fig 11, with a range of roughly 4,000–8,000 overall mortality. The number of overall infections do not change as much along the Pareto front, with an overall difference of only 70, 000 infections between the two ends of the front. We further find that the mortality minimizing allocation at right end of the Pareto front yields only a marginal reduction in mortality compared to an allocation in the middle of the Pareto front. This observation suggests the choice of an allocation in the middle of the Pareto front (marked by dash-dotted gray line) which corresponds to an allocation of 60% of the vaccines to age group 30–39, roughly 13% to age group 60–69 and to age group 80, and then the rest to age groups 70–79 and 20–29. This result provides a snapshot of the weak-spots of the Israeli vaccine coverage. For example, it reflects the fact that, at the time of submission, the vaccine immunity of nearly 18% of age group 30–39 has waned and expired since they did not take a booster dose.

Under current assumptions, the optimal allocation for the scenario considered here does not include the vaccination of those under 20. This stems from vaccine immunity weak spots in other age groups, and from the assumptions that the relative susceptibility of children under the age of 20 is lower than that of older ages. This does not imply that vaccinating children is not beneficial, nor does it imply that the vaccination of children in Israel is not recommended. It does imply that the weakest spots in vaccine coverage in Israel are in age groups older than 20. Hence if one needs to choose between the investment of efforts in the vaccination of children aged 5–9 or their parents, then at the time of evaluation, the vaccination of the latter group yields better epidemic outcomes.

The vast majority of random allocations give rise to outcomes that are significantly worse than those that arise from allocations along the Pareto front. For example, we observe that a uniform allocation of vaccines is far from the Pareto front and results in 1.95 million additional infections and 8,600 overall mortality. This implies that, at this stage, precision in implementing the optimal allocation is important, and that it is necessary to re-evaluate the optimal allocations as the vaccination effort continues.

The above computation illustrates the broad applicability of the tools developed in this work. We note, however, that the computation above does not take into account estimates of those who recovered from SARS-CoV-19 but were never detected. It also does not account for further waning of immunity. Finally, already during the time of submission conditions have started to change with the emergence of a new variant (which later became known as the omicron variant). Therefore, applying such a computation to guide decision making in Israel will require refinement and update.

## 5 Discussion

This study uses modelling and optimization to explore the outcomes of vaccination campaigns for SARS-CoV-19, with emphasis on the effects of vaccinating children. We demonstrate the use of Pareto front computations to systematically evaluate the trade-offs involved among conflicting measures for optimizing vaccine allocations such as mortality and attack rate. In particular, we utilize this approach to compare optimal achievable outcomes when all age-groups can be vaccinated to those that can be attained when younger age groups are not eligible for vaccination.

Our study focused on two questions:

1. How essential is the vaccination of children and youths to achieving herd immunity?
2. What is the population level impact of vaccination of children in case herd immunity by vaccination cannot be attained?

Regarding the question of herd immunity, we estimated that when ages 10 and older are eligible for vaccination, optimal allocations can lead to herd immunity for basic reproduction numbers up to $R_{\text{critical}}^{10+}\approx 3.5$. Though this threshold varies (±20%), e.g., across countries and in dependence on vaccine efficacy and prior immunity, we can conclude that at *R*_0_ values estimated for the currently circulating variants [37], herd immunity can most probably not be achieved without vaccination of children under 10, even if performed in an optimal way. More fundamentally, our results show that designing vaccine allocation with the aim of achieving herd immunity is not a robust strategy. Indeed, we show that allocations optimized to achieve herd immunity give preference to the young and leave the older age groups exposed. Consequently, if, due to a mis-estimation or to changed circumstances, herd-immunity is not achieved, mortality would rise steeply.

The second question concerns the cases in which the ‘herd immunity’ strategy is not feasible. We find that in most cases considered, age groups 0–9 and 10–19 are included in the optimal allocations, yet the degree to which their inclusion affects epidemic outcomes varies by case. The exclusion of age groups 0–9 or 0–19, i.e., allocation of the same number of vaccine to other ages, only marginally affects outcomes in terms of mortality. The exclusion of age group 10–19 results in significantly worse outcomes in terms of the minimal number of infections achievable. Finally, the inclusion of age group 0–9 leads to a reduction in the minimal number of infections possible at high *R*_0_.

We should stress here that the above results, which demonstrate in some cases a limited improvement in outcomes with the inclusion of children, should not be taken to imply that vaccination of children is not in itself beneficial and important. The comparisons here are performed under the assumption of a fixed supply of vaccines, in which case vaccination of one age group entails an opportunity cost in not vaccinating another. Obviously if one can extend the coverage so as to include children, without reducing vaccination levels in other age groups, then doing so will only improve outcomes. Indeed our results emphasize the importance of increasing total vaccination coverage to the highest extent possible, in that they demonstrate the limitations of what can be achieved with a limited amount of vaccine, even if it is optimally allocated.

Most examples provided in the paper consider the design of a new vaccination campaign, while focusing on the case in which there is a shortage of vaccines, hence a need to optimize their allocation. However, the methodology presented in this work applies more broadly at later stages of a vaccination campaign, taking into account those already vaccinated, and in cases where vaccine shortage is not the key limiting factor. Indeed, we provide an example which demonstrates the use of the tools to reveal the weak spots of the current vaccination coverage in Israel, e.g., with the aim of better focusing a public awareness campaign to the appropriate age groups. This example also demonstrates the importance of considering the trade-offs between mortality and infections, and shows the Pareto front allows one to evaluate those trade-offs and make an informed choice of the allocation policy. Finally, the Pareto front allows to assess the cost of sub-optimal allocations and to determine the degree to which precision in implementing the chosen allocation is important.

This study is subject to several limitations. We focus on the outcomes in the medium-term range after the vaccination campaign has ended. Over longer timescales, the possibility of virus mutation will influence these predictions. Similarly, if immunity is not maintained by booster vaccination, long term predictions will be influenced by waning immunity. Our study optimizes outcomes for the post-vaccination phase, and is, therefore, most relevant when disease spread is contained during the vaccination campaign, e.g., by non-pharmaceutical interventions. In this case, once a vaccine allocation that is optimized for post-vaccination outcomes is determined, transient features of a vaccination campaign that results in the desired allocation can be designed, for example, to allow gradually relaxing non-pharmaceutical interventions during the campaign [21]. In case the vaccination campaign occurs in parallel to an ongoing outbreak, short-term goals are likely to dominate the design of the vaccination campaign [22]. We have also used pre-pandemic contact matrices in accordance with the aim of returning to pre-pandemic routine after the vaccination campaign. Nevertheless, we present results for a range of basic reproduction numbers *R*_0_, and therefore implicitly account for a new routine which might include a degree of non-pharmaceutical interventions. However, age-dependent non-pharmaceutical interventions such as long-term changes in school operation are not well captured by this approach. Accounting for such interventions will require the estimation and application of post-pandemic contact matrices.

The debate over childrens’ vaccination is on-going in many countries, and will reemerge as booster vaccinations or lineage-adapted vaccines will be considered. More generally, the management of vaccination campaigns will continue to require making decision based on the population-level impact of various alternatives. We believe that the approach presented in this work can provide valuable model-informed tools to assist such decision making.

## Supporting information

S1 Text Impact of changes in parameters. Section S1.1: Changes in contact matrix. Section S1.2: Results for all-or-none vaccines. Section S1.3: Impact of vaccine efficacy. Section S1.4: Impact of vaccine coverage. Section S1.5: Impact of vaccine hesitancy. Section S1.6: The effect of preexisting immunity in the population due to recovery.
(PDF)

S1 Fig Effect of perturbations in contact matrix on allocations along Pareto fronts. (EPS)

S2 Fig Pareto front with all-or-none vaccine. (EPS)

S3 Fig Final size of epidemic with all-or-none vaccine. (EPS)

S4 Fig Impact of change in reproduction number with all-or-none vaccines. (EPS)

S5 Fig Effect of vaccine efficacy in blocking transmission (reducing susceptibility) onvaccination coverage required for herd immunity. (EPS)

S6 Fig Effect of vaccine efficacy on critical reproduction numbers. (EPS)

S7 Fig Pareto fronts for different values vaccine efficacy. (EPS)

S8 Fig Pareto fronts for different vaccine coverage. (EPS)

S9 Fig Effect of vaccine hesitancy on critical reproduction numbers. (EPS)

S10 Fig Pareto fronts for different vaccine hesitancy levels. (EPS)

S11 Fig Impact of preexisting immunity on vaccination coverage required for herd immunity. (EPS)

S12 Fig Impact on preexisting immunity on critical reproduction numbers. (EPS)

S13 Fig Pareto front with preexisting immunity. (EPS)
