# Supplementary material for: The role of childrens’ vaccination for COVID-19—Pareto-optimal allocations of vaccines
Source: PLoS Comput Biol. 2022 Feb 25;18(2):e1009872. doi: 10.1371/journal.pcbi.1009872 (PMC8906616; doi:10.1371/journal.pcbi.1009872)
Supplement: S1 Text — Section S1.1: Changes in contact matrix. Section S1.2: Results for all-or-none vaccines. Section S1.3: Impact of vaccine efficacy. Section S1.4: Impact of vaccine coverage. Section S1.5: Impact of vaccine hesitancy. Section S1.6: The effect of preexisting immunity in the population due to recovery. (PDF) [file pcbi.1009872.s001.pdf]

The role of childrens' vaccination for COVID-19 - Pareto-optimal allocations of vaccines
Supporting information

Nir Gavish[1*], Guy Katriel[2]

1 Faculty of Mathematics, Technion - IIT, Haifa, Israel
2 Department of Applied Mathematics, ORT Braude College of Engineering, Karmiel, Israel

* ngavish@technion.ac.il

S1 Impact of changes in parameters

We consider the impact of changes in assumptions concerning, e.g,. vaccine efficacy or vaccine hesitancy.

Note that any reference to an equation in the following refers to an equation in the main text.

S1.1 Changes in contact matrix

The contact matrices reflect demography and the level of interactions among age groups in the population under study. Since estimation of contact matrices involves statistical uncertainties, and since interaction patterns vary spatially (e.g. urban vs rural areas), it is important to evaluate the sensitivity of the optimal allocations along the Pareto front to changes in the contact matrix. To do so, we randomly modify the level of interaction of each age group by 10%. Namely, we draw uniform noise $(x_1, x_2, \cdots, x_m) \sim U([0.9, 1.1]^m)$ and define the perturbed contact matrix as

$$C_{ij}^{\text{perturbed}} = DC_{ij}D, \quad D = \mathrm{diag}(\mathrm{x}_1, \mathrm{x}_2, \cdots, \mathrm{x}_\mathrm{m}).$$

We then compute the Pareto front corresponding to the perturbed contact matrix. Fig S1 presents the optimal allocations per age group along the Pareto front corresponding to C_{ij} (red solid curve), as well as Pareto front allocations corresponding to 40 randomly perturbed contact matrices (blue dots). We observe that the optimal allocations are generally insensitive to noise. One effect of noise is that the ends of the Pareto fronts are shifted. For example, the infections-minimizing end of the perturbed Pareto front varies in the of 154M-172M. This range reflect a ±7% change from the outcomes of the infection minimizing allocation of the non-perturbed Pareto front with 165M overall infected, in accordance with the 10% noise level introduced to the contact matrices. Similarly, we find that transition points between different vaccine allocations are shifted by a smaller or comparable level, see, e.g., age group 40-49 or 70-79. Particularly, the allocation of vaccines in age group 0-9 is characterized by many transitions along the Pareto front and therefore we observe that it is more effected by noise.

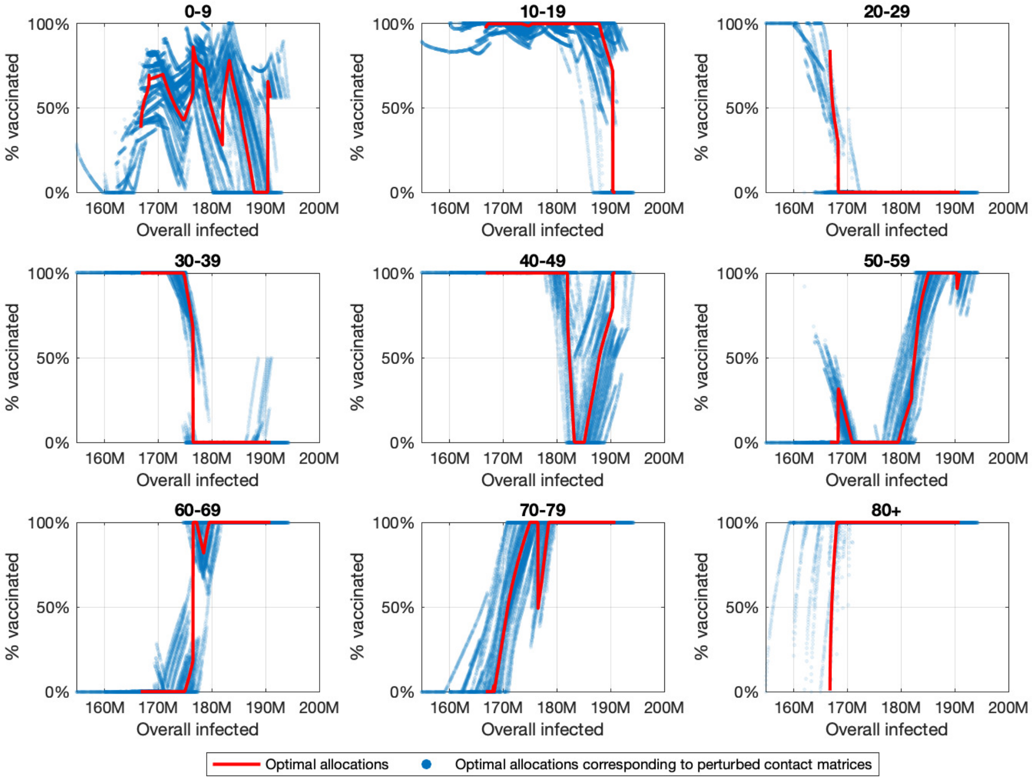

Fig S1: **Effect of perturbations in contact matrix on allocations along Pareto fronts.** Red solid curves are the Pareto-optimal vaccine allocations per age group along the Pareto front presented in Fig 6 in the main text when all ages are eligible for vaccination. Blue markers are Pareto-optimal vaccine allocations corresponding to an overall of 40 perturbed contact matrices with a 10% noise level.

S1.2 Results for all-or-none vaccines

We allow for the possibility that a fraction $1-\nu$ of the population vaccinated does not generate immunity, while the rest of the vaccinated population is fully immune ($\varepsilon = 0$). This case is known as 'all-or-none' vaccine, whereas the case in which all vaccinated population are partially immune ($0 < \varepsilon < 1$) corresponds to the case of a 'leaky' vaccine.

The computation of herd immunity thresholds is identical in the 'leaky' or 'all-or-none' cases. Indeed, these computations rely on the absolute value of the dominant eigenvalue of (7), and thus their dependence on ν and ε is only through the expression $\epsilon\nu$. However, when herd immunity is not reached, leaky vaccines are known to result in a higher prevalence of infection than 'all-or-none' vaccines [1]. Indeed, for the USA example presented in Fig 5 in the main text, we observe that allocations along the Pareto front corresponding to 'all-or-none' vaccines give rise to 20% less infections than allocations along the Pareto front corresponding to 'leaky' vaccines, see Fig S2. Details of the outcomes of a vaccination campaign with 'all-or-none' vaccines are presented in Fig S3. In cases A and B of Fig S3, the reductions in overall infections are 28% and 25%, respectively. Finally, we examine the impact of the nature of protection on optimal allocations as the reproduction number changes. Allocations minimizing infections are nearly identical in the two cases when R_0 is in the lower range, but for higher R_0, while the leaky case displays the transition in which children of ages 0-9 enter the allocation, as discussed in the text, no such phenomenon occurs in the all-or-none case.

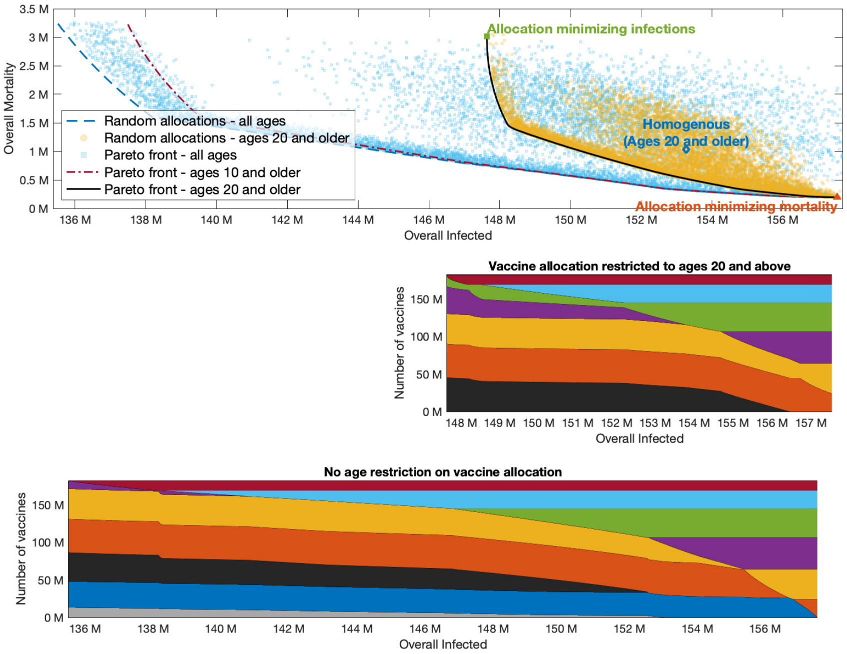

Fig S2: **Pareto front with all-or-none vaccine.** Same as Fig 5 in the main text, but with 90% all-or-none vaccines.

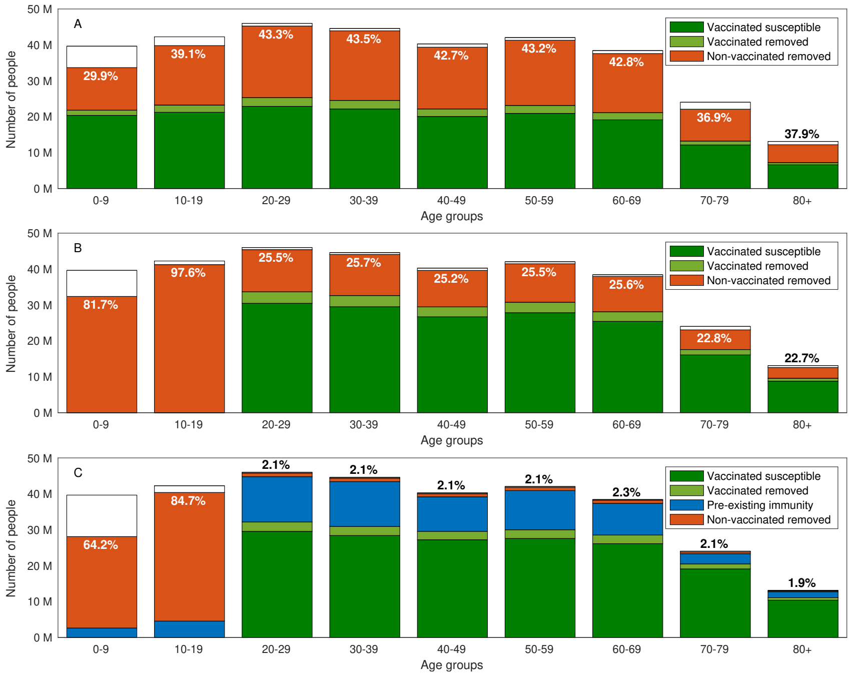

Fig S3: **Final size of epidemic with all-or-none vaccine.** Same as Fig 1 in the main text but with all-or-none vaccines: The expected outcome of a partial return to normality in the USA to a basic reproduction number of $R_0 = 3$ after completion of a vaccination campaign covering 55% of the population. Removed population refers to those recovered or dead. The computation considers a vaccination campaign in which A: All the population is eligible for vaccinations. B: Vaccine eligibility is limited to ages 20 and above. C: Vaccine eligibility is limited to ages 20 and above, and at the time normality is restored 20% of the population is recovered from COVID-19, and the prevalence of active cases is 0.5% of the population. The text in all graphs corresponds to the percent of non-vaccinated removed individuals in each age group.

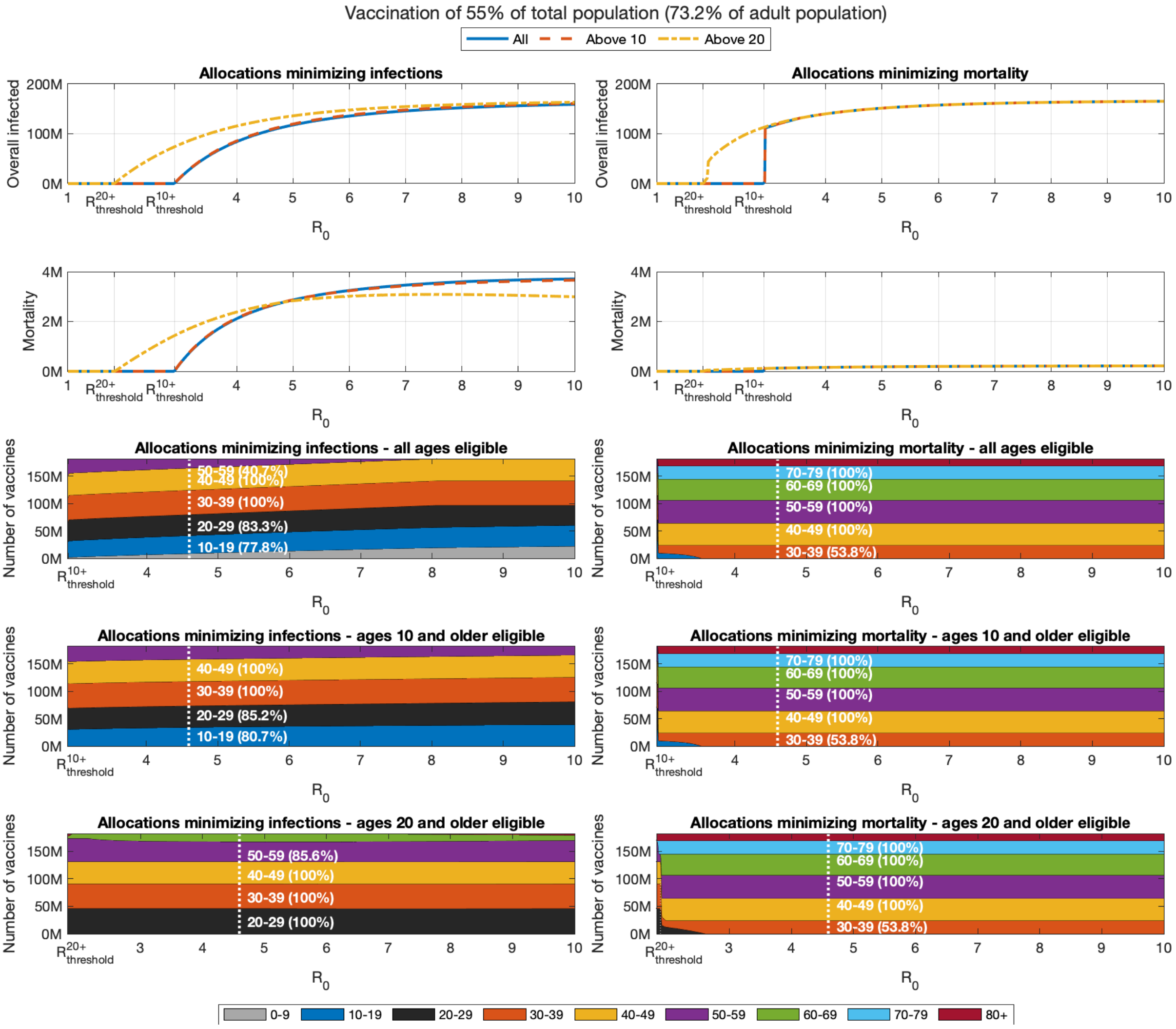

Fig S4: **Impact of change in reproduction number with all-or-none vaccines** Same as Fig 4 in the main text, but with 90% all-or-none vaccines.

S1.3 Impact of vaccine efficacy

We consider the impact of vaccine efficacy in reducing the susceptibility of those vaccinated on the outcomes of the vaccination campaign.

The relative susceptibility ε of vaccinated individuals effects the vaccine supply thresholds and the critical reproduction numbers solely through the expression εp_i in (7), were p_i is the portion of age group i that is vaccinated. Therefore, in terms of vaccine supply thresholds and the critical reproduction numbers, changes in vaccine efficacy are equivalent to changes in vaccination coverage. As expected, increase of vaccine efficacy lowers the vaccine supply threshold required for herd immunity and vice-versa, see Fig S5 in main text. Particularly, children under the age of 10 appear in the allocation that achieves herd immunity with minimal converge at higher values of R_0. Similar behavior is observed in computations

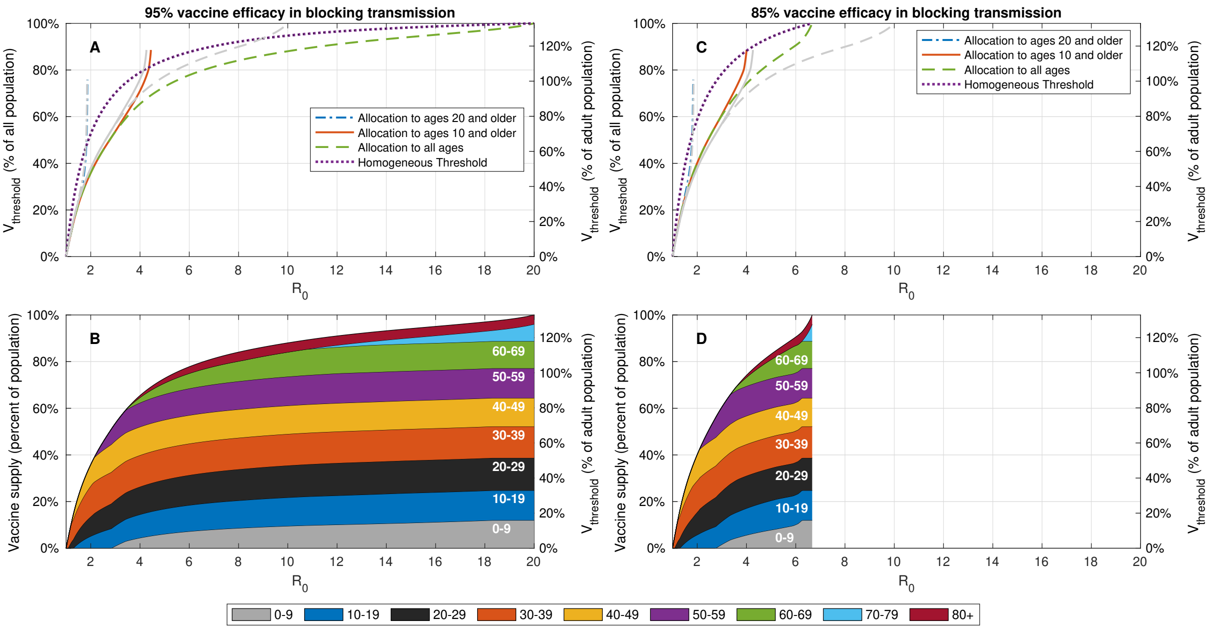

Fig S5: **Effect of vaccine efficacy in blocking transmission (reducing susceptibility) on vaccination coverage required for herd immunity.** A,C: Vaccine coverage $V_{\text{threshold}}$ required to achieve herd immunity threshold as a function of the reproduction number R_0 for the USA demography and contact structure. The gray curves correspond to the case of 90% vaccine efficacy. B,D: Vaccine allocations at which herd immunity is achieved at minimal vaccine coverage and when all the population is eligible for vaccination.

adapted for various countries. Particularly, we find that a change of 5% in vaccine efficacy shifted the critical reproduction number $R^{10+}_{\text{critical}}$ by $\Delta R_0 = 0.25$ on average, see Fig S6. However, when only ages 20 and older are eligible for vaccination, the shift in the critical reproduction number $R^{20+}_{\text{critical}}$ is much smaller.

We further consider the impact of vaccine efficacy in cases in which herd immunity is not achieved. As expected, the optimal outcomes that result from optimal vaccine distribution along the Pareto front improve with an increase in vaccine efficacy. We observe that a 5% change in vaccine efficacy leads to roughly 33% change in the minimal overall mortality that can be achieved, and 40%-70% change in the minimal overall infections that can achieved by proper vaccine allocation, see Fig S7. Moreover, we observe that as vaccine efficacy decreases, the optimal vaccine allocation shifts toward the vaccination of younger ages.

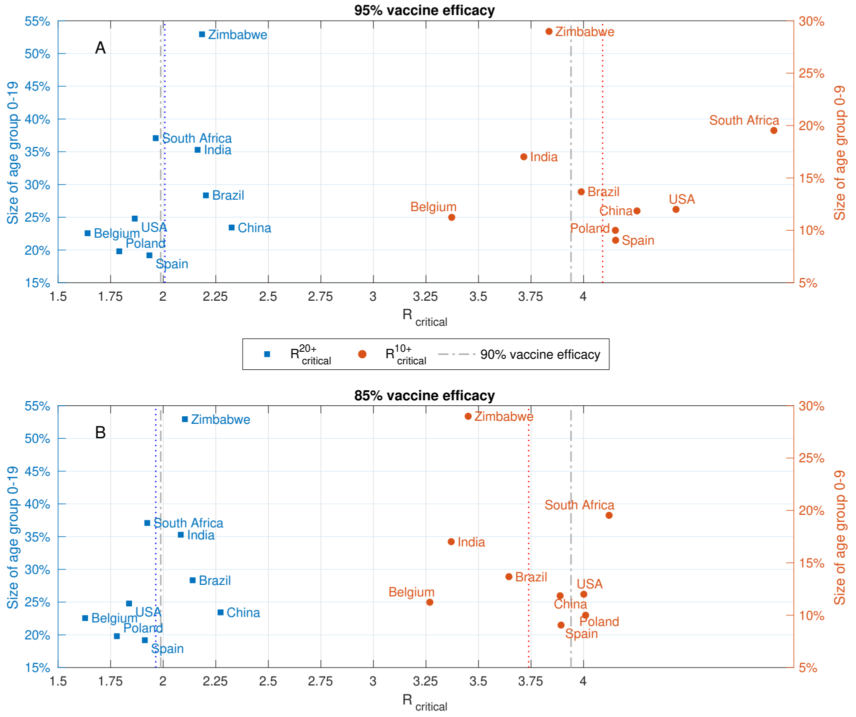

Fig S6: **Effect of vaccine efficacy on critical reproduction numbers.** Reproduction numbers $R^{20+}_{\text{critical}}$ and $R^{10+}_{\text{critical}}$ at which herd immunity cannot be achieved without vaccination of age groups $0-19$ and $0-9$, respectively. Computed for A: 95% vaccine efficacy in blocking transmission. B: 85% vaccine efficacy in blocking transmission. The gray curves in both plots correspond to the case of 90% vaccine efficacy.

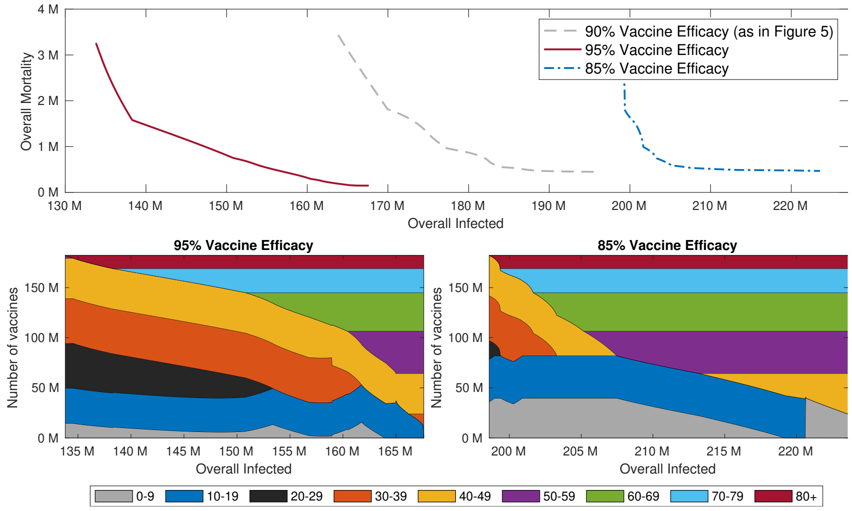

Fig S7: **Pareto fronts for different values vaccine efficacy.** Top graphs: Pareto fronts corresponding to 95% (solid red), 90% (dashed gray) and 85% vaccine efficacy (dash-dotted blue). All other parameters are as in Fig 1 in main text. Bottom Graphs: Vaccine allocations along the corresponding Pareto fronts with 85% vaccine efficacy (left) and 75% vaccine efficacy (right).

S1.4 Impact of vaccine coverage

When herd immunity is not achievable, vaccine coverage becomes a key parameter in the design of a vaccination campaign. As expected, the optimal outcomes that result from optimal vaccine distribution along the Pareto front improve with an increase in vaccine coverage. We observe that a 3% change in vaccine coverage (from the baseline of 55%) leads to roughly 6% change in the minimal overall mortality that can be achieved, and 11% change in the minimal number of infections that can achieved by proper allocation of vaccines, see Fig S8. The reason for the relatively small change in overall mortality is that

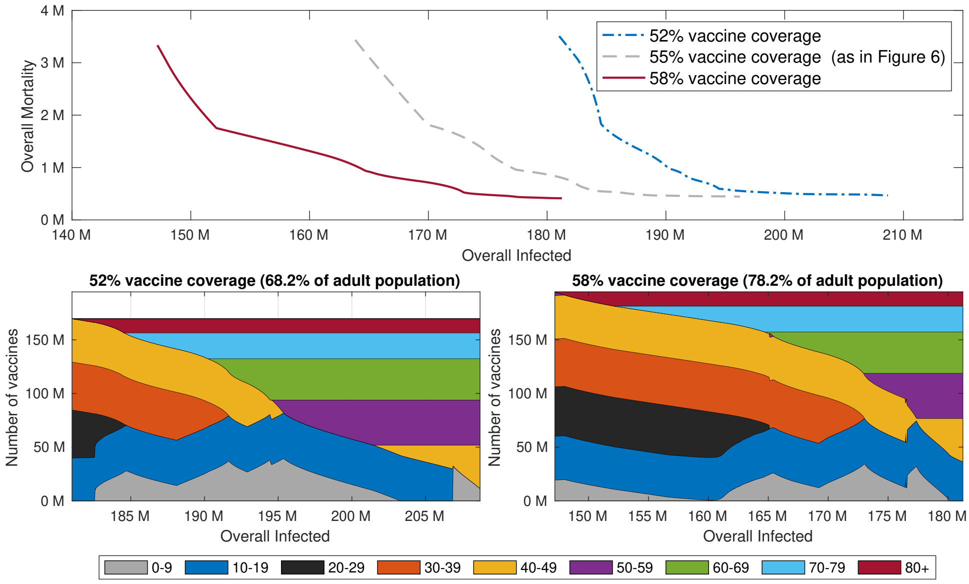

Fig S8: **Pareto fronts for different vaccine coverage.** Top graphs: Pareto fronts corresponding to 52% (solid red), 55% (dashed gray) and 58% vaccine coverage (dash-dotted blue). All other parameters are as in Fig 5 in main text. Bottom Graphs: Vaccine allocations along the corresponding Pareto fronts with 52% vaccine coverage (left) and 58% vaccine coverage (right).

already at relatively low vaccine coverage the optimal vaccine allocation for minimizing mortality spans the older age groups, and additional vaccine coverage is mostly utilized to extend allocations to younger age groups which provide indirect protection.

S1.5 Impact of vaccine hesitancy

The examples presented in Figs 1-7 in the main text all consider cases where vaccine allocations can include vaccination of 100% of an age group. However, vaccine hesitancy, logistical difficulties and existing medical conditions are likely to limit actual vaccination coverage within an age group.

The constraint that vaccine allocation cannot exceed 90% of each age group leads to a significant reduction in the critical reproduction number $R^{10+}_{\text{critical}}$ for which herd immunity is achievable by proper allocation of vaccines from an average of $R^{10+}_{\text{critical}} \approx 3.6$ in various countries to $R^{10+}_{\text{critical}} \approx 3$, see Fig S9. Imposing a stricter constraint that vaccine allocation cannot exceed 80% of each age group, leads to a further reduction to $R^{10+}_{\text{critical}} \approx 2.5$.

We observe that limiting vaccine coverage per age group to 90% results in an 220% increase in the minimal overall mortality that can be achieved and that further limiting vaccine coverage per age group to 80% results in an 330% increase in the minimal overall mortality that can be achieved, see Fig S10. These results strongly suggest that a key performance measure for the success of a vaccination campaign in reducing mortality should be vaccine coverage per age group, particularly in older age groups. In addition, we observe that the minimal number of infections that can achieved by proper allocation of vaccines is not as sensitive to the maximal possible vaccine coverage per age group. Indeed, we find that limiting vaccine coverage per age group to 90% results in 7% increase in the minimal overall mortality

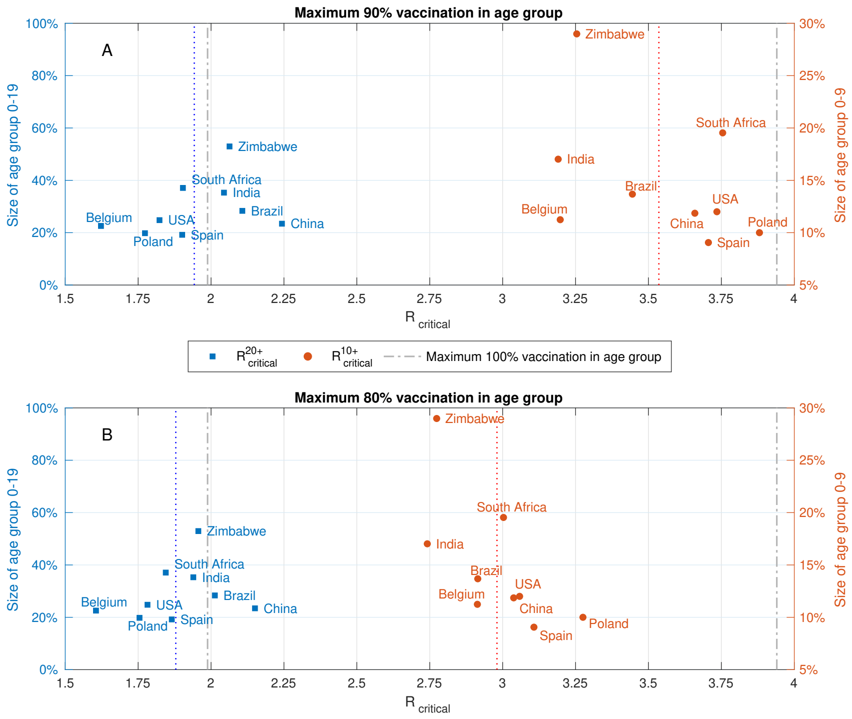

Fig S9: **Effect of vaccine hesitancy on critical reproduction numbers.** Reproduction numbers $R^{20+}_{\text{critical}}$ and $R^{10+}_{\text{critical}}$ at which herd immunity cannot be achieved without vaccination of age groups $0-19$ and $0-9$, respectively. Computed for A: Maximum 90% vaccination in age group. B: Maximum 80% vaccination in age group. The gray curves in both plots correspond to the case when there is no constraint on the number of vaccines that can be allocated to an age group.

that can be achieved and that further limiting vaccine coverage per age group to 80% results in an 19% increase.

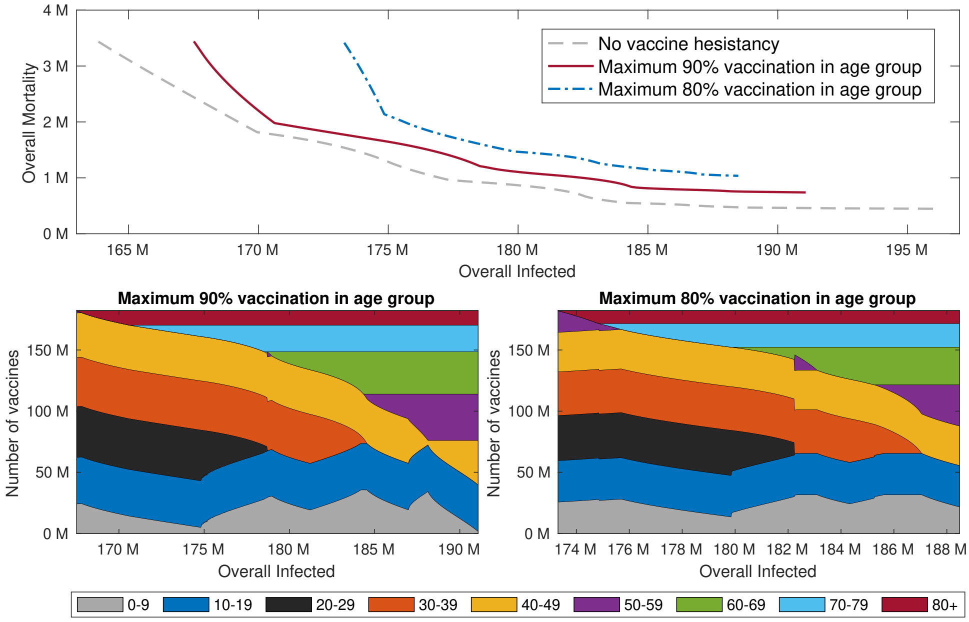

Fig S10: **Pareto fronts for different vaccine hesitancy levels.** Top graphs: Pareto fronts corresponding to a maximum of 90% (solid red), 100% (dashed gray) and 80% vaccine coverage per age group (dash-dotted blue). All other parameters are as in Fig 5 in the main text. Bottom Graphs: Vaccine allocations along the corresponding Pareto fronts with 90% maximal vaccine coverage per age group (left) and 80% maximal vaccine coverage per age group (right).

S1.6 The effect of preexisting immunity in the population due to recovery

Most results presented in this work concerned a population which is fully susceptible, unless vaccinated, at the end of the vaccination campaign. In practice, as of December 2021, the number of COVID-19 cases exceeded 260 million globally [2], most of whom are recovered. We now examine the effect of preexisting immunity in the population due to recovery. In particular, we consider cases in which 10% or 20% of the population are recovered and immune at the end of the vaccination campaign. We determine the distribution of the recovered cases among age groups in a way which is roughly equivalent to running a simulation without any vaccination until the recovered compartments reach the desired size. This is done by determining the distribution of the recovered cases according to the dominant eigenvector of the next generation matrix. A recovered individual is assumed to be fully immune to re-infection. Note, in comparison, that vaccinated individuals are assumed to be 90% immune to infection. In the computations below, we further assume that vaccines are not allocated to recovered cases.

We first consider the impact of preexisting immunity on vaccination coverage required for herd immunity. As expected, the leading order effect of 10% or 20% preexisting immunity, is that vaccine coverage $V_{\text{threshold}}$ required to achieve herd immunity is reduced by 12% or 25%, respectively, see Fig S11. The differences between the reduction in $V_{\text{threshold}}$ and the percent of population with preexisting immunity stem from the difference in the assumed immunity of recovered and vaccinated, and the fact that preexisting immunity is not optimally distributed.

As expected, the critical reproduction numbers at which herd immunity cannot be achieved without vaccination of age groups $0-9$ or $0-19$ increase with the percent of the individuals with preexisting immunity in these age groups. We compute the critical reproduction numbers for nine different countries, and observe that on average, prior immunization of 10% of the population results in the increase of roughly 7% in the critical reproduction numbers $R^{10+}_{\text{critical}}$ and $R^{20+}_{\text{critical}}$. Similarly, prior immunization of 20% of the population results in an increase of about 15% in the critical reproduction numbers $R^{10+}_{\text{critical}}$ and $R^{20+}_{\text{critical}}$.

When comparing pre-existing immunity to immunity induced by vaccines, one has to take into account that the distribution pre-existing immunity is determined by epidemic dynamics and therefore cannot

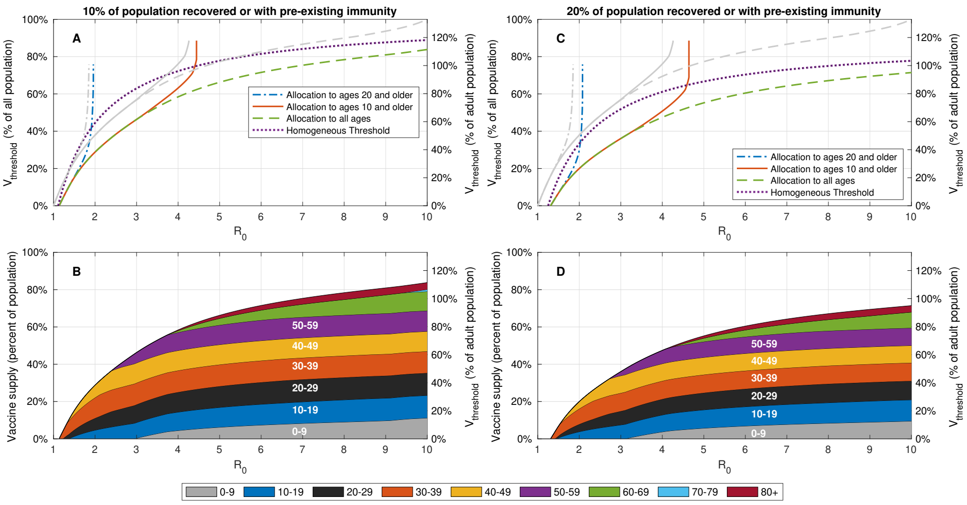

Fig S11: **Impact of preexisting immunity on vaccination coverage required for herd immunity.** A,C: Vaccine coverage $V_{\text{threshold}}$ required to achieve herd immunity threshold as a function of the reproduction number R_0 for the USA demography and contact structure. The gray curves corresponds to the case of no preexisting immunity. B: Vaccine allocations at which herd immunity is achieved at minimal vaccine coverage and when the entire population is eligible for vaccination.

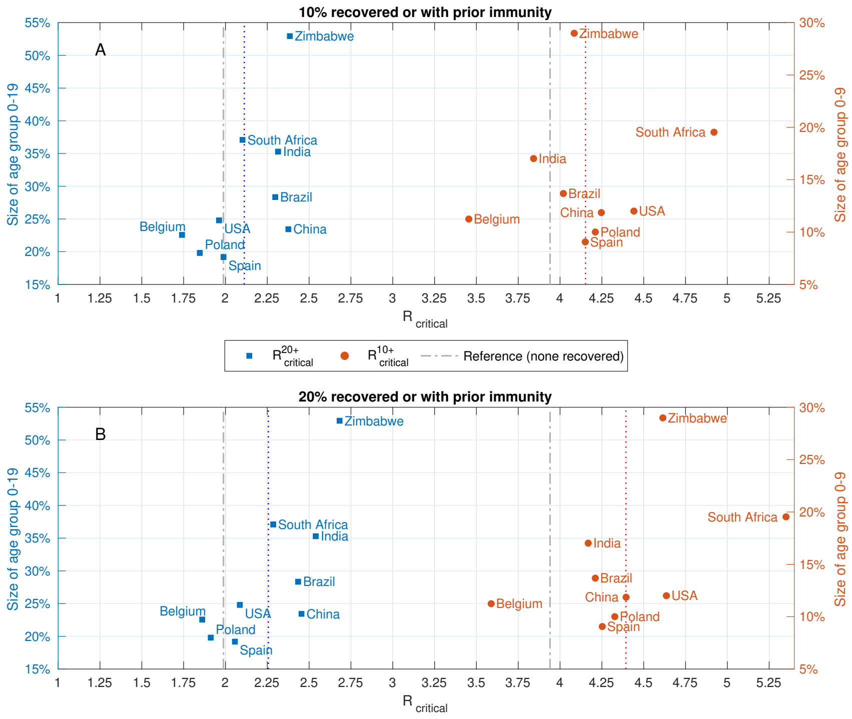

Fig S12: **Impact on preexisting immunity on critical reproduction numbers.** Reproduction numbers $R^{20+}_{\text{critical}}$ and $R^{10+}_{\text{critical}}$ at which herd immunity cannot be achieved without vaccination of age groups $0-19$ and $0-9$, respectively. Computed for A: 10% of the population is recovered or has prior immunity. B: 20% of the population is recovered or has prior immunity. The gray curves in both plots correspond to the case when there is no prior immunity.

be optimized. On the other, it is assumed that vaccine induced immunity provides partial protection while convalescent immunity provide full protection. In what follows, we aim to understand the impact of

pre-existing immunity when the overall portion of the protected population does not change, namely the portion of vaccinated or recovered population is constant.

We now consider the case in which 20% of the population is recovered or has prior immunity and vaccine coverage is 35% of the population, and compare it to the case in which vaccine coverage is 55% of the population and preexisting immunity does not exists. We observe that in the case of preexisting immunity the number of infections at the Pareto front increase by roughly 10-15% compared to a case of no preexisting immunity, and mortality increases by 20-30%, see Fig S13. This difference stems from the fact that preexisting immunity is not optimally distributed. We note that in this case we reduced vaccine coverage to keep the total portion of the protected population constant to allow comparison. In case vaccine coverage is not reduced, pre-existing immunity would obviously lead to much better epidemic outcomes.

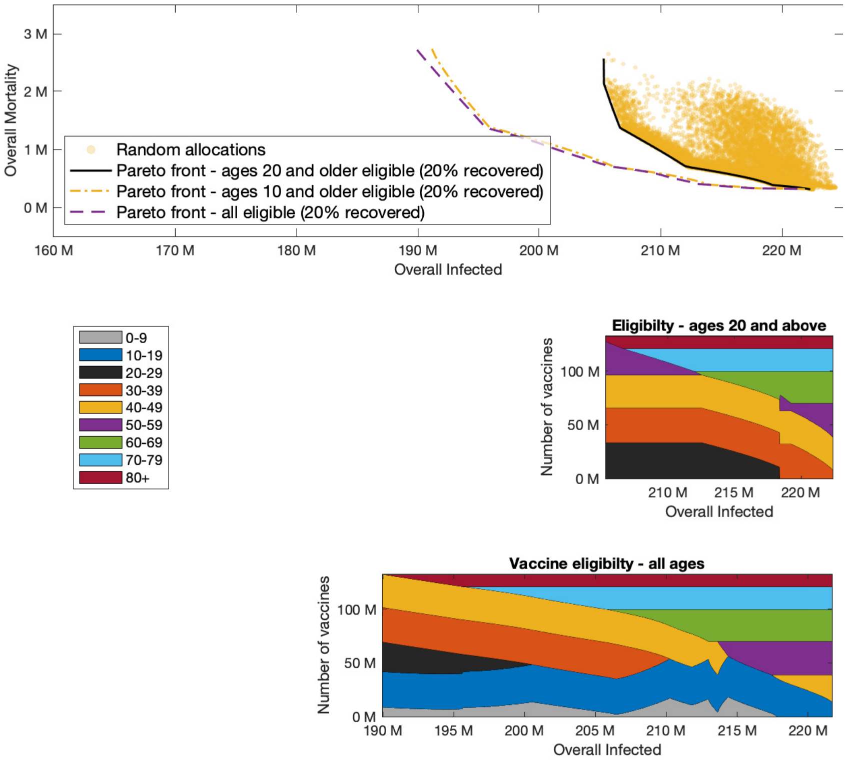

Fig S13: **Pareto front with preexisting immunity.** Pareto fronts computed for the case in which 20% of the population is recovered or has prior immunity and vaccine coverage is 35% of the population.

References

1. Magpantay FM, Riolo MA, De Celles MD, King AA, Rohani P. Epidemiological consequences of imperfect vaccines for immunizing infections. SIAM Journal on Applied Mathematics. 2014;74(6):1810–1830.

2. WHO Coronavirus (COVID-19) Dashboard. https://covid19whoint/. 2021;.
